# Structural Analysis of Monomeric RNA-Dependent Polymerases: Evolutionary and Therapeutic Implications

**DOI:** 10.1371/journal.pone.0139001

**Published:** 2015-09-23

**Authors:** Rodrigo Jácome, Arturo Becerra, Samuel Ponce de León, Antonio Lazcano

**Affiliations:** 1 Facultad de Ciencias, Universidad Nacional Autónoma de México, Cd. Universitaria, México D.F., México; 2 Dirección General de Investigación, Facultad de Medicina, Universidad Nacional Autónoma de México, Cd. Universitaria, México D.F., México; 3 Miembro de El Colegio Nacional, México D.F., México; Division of Clinical Research, UNITED STATES

## Abstract

The crystal structures of monomeric RNA-dependent RNA polymerases and reverse transcriptases of more than 20 different viruses are available in the Protein Data Bank. They all share the characteristic right-hand shape of DNA- and RNA polymerases formed by the fingers, palm and thumb subdomains, and, in many cases, “fingertips” that extend from the fingers towards the thumb subdomain, giving the viral enzyme a closed right-hand appearance. Six conserved structural motifs that contain key residues for the proper functioning of the enzyme have been identified in all these RNA-dependent polymerases. These enzymes share a two divalent metal-ion mechanism of polymerization in which two conserved aspartate residues coordinate the interactions with the metal ions to catalyze the nucleotidyl transfer reaction. The recent availability of crystal structures of polymerases of the Orthomyxoviridae and Bunyaviridae families allowed us to make pairwise comparisons of the tertiary structures of polymerases belonging to the four main RNA viral groups, which has led to a phylogenetic tree in which single-stranded negative RNA viral polymerases have been included for the first time. This has also allowed us to use a homology-based structural prediction approach to develop a general three-dimensional model of the Ebola virus RNA-dependent RNA polymerase. Our model includes several of the conserved structural motifs and residues described in other viral RNA-dependent RNA polymerases that define the catalytic and highly conserved palm subdomain, as well as portions of the fingers and thumb subdomains. The results presented here help to understand the current use and apparent success of antivirals, i.e. Brincidofovir, Lamivudine and Favipiravir, originally aimed at other types of polymerases, to counteract the Ebola virus infection.

## Introduction

**Fig 1:**
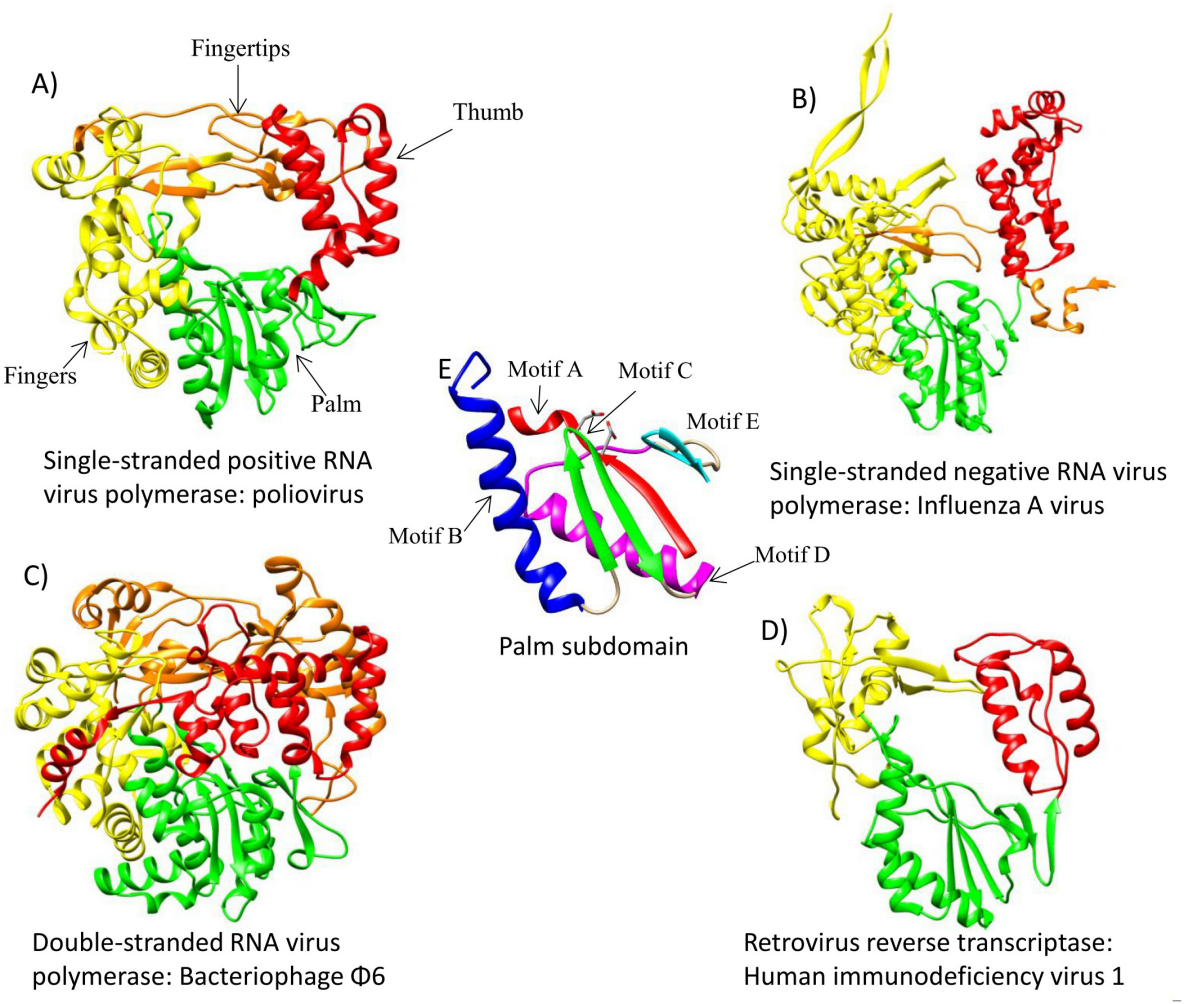
Three dimensional structures of the polymerases of the four main RNA viruses groups. In the center of the figure the palm subdomain present in all of the polymerases is shown. (A) single-stranded positive RNA virus: poliovirus RNA-dependent RNA polymerase (PDB code: 3OL6); (B) single-stranded negative RNA virus: influenza A virus RNA-dependent RNA polymerase (PDB code: 4WSB); (C) double-stranded RNA virus: bacteriophage Φ6 RNA-dependent RNA polymerase (PDB code: 1HHS); (D) reverse-transcribing virus: HIV-1 reverse transcriptase (PDB code: 3DLK). (E) The highly conserved palm subdomain showing the position of the conserved catalytic aspartic acid residues (edited from 3DLK). The color code for the subdomains is fingers subdomain, yellow; palm subdomain, green; thumb subdomain, red; fingertips, orange. The conserved structural motifs in the palm subdomain are colored as follows: motif A, red; motif B, dark blue; motif C, green; motif D, magenta; motif E, cyan.

Due to their role in replication, transcription, and reverse transcription in the case of reverse-transcribing viruses, RNA-dependent RNA polymerases (RdRp) and reverse transcriptases (RT) are key enzymes in the viral biological cycle. Following the crystallization of the poliovirus RdRp by Hansen et al. [1], over 20 distinct viral RNA polymerases crystals have been obtained which belong to single-stranded positive RNA (ss(+)RNA) viruses of the families Flaviviridae, Picornaviridae, Caliciviridae and Leviviridae; single-stranded negative RNA (ss(-)RNA) viruses of the families Orthomyxoviridae and Bunyaviridae; double-stranded RNA (dsRNA) viruses of the families Reoviridae, Cystoviridae and Birnaviridae; and reverse transcribing viruses of the family Retroviridae. They are all part of the superfamily of DNA- and RNA polymerases, which are characterized by a right hand architecture with three functional subdomains, i.e. fingers, palm and thumb; and a two metal ion mechanism of action in which two aspartic acid residues located in the palm subdomain interact with two divalent metal ions to achieve the nucleophilic attack, which allows the incorporation of the incoming ribonucleotide to the RNA chain [2,3]. The primary structure of RdRps and RTs is characterized by the sequence fingers-palm-fingers-palm-thumb, and in the tertiary structures of the former there are several extensions from the fingers, named “fingertips”, that extend towards the thumb subdomain, giving the appearance of a closed right-hand shape (Fig 1), in contrast with the U-shaped form of RTs and of DNA-dependent DNA polymerases of the families A, B and Y (Fig 1) [4]. The palm subdomain is the catalytic subdomain and is by far the most conserved region of all monomeric viral RNA polymerases. It is formed by a β-sheet with three to six β-strands that lie above two helices, and has the conserved catalytic aspartic acid residues that coordinate the two metal ions necessary for the phosphoryl transfer reaction (Fig 1E) [5,6]. It has been hypothesized that the palm subdomain is the oldest domain of these enzymes, and that it may be a relic of an RNA/protein world that existed prior to the evolution of cellular DNA genes [7–9]. The fingers subdomain is a mixed α/β structure that plays a key role in the interactions with the template strand and the incoming nucleotide. The thumb subdomain is a highly variable subdomain with a predominantly helical structure located opposite the fingers that forms non-specific interactions with the primer strand.

Six conserved structural motifs (motifs A-F) have been identified in the tertiary structures of RdRps and RTs [3–5,10,11]. With the exception of motif F [12], which is located in the fingers subdomain, motifs A-E are all located in the palm subdomain (Fig 1E). Additional structural motifs and functional regions have been identified in some polymerase subgroups such as motifs G [13,14] and H of ss(+) and dsRNA viruses [10], or motifs G and H of ss(-)RNA viruses [15]. All these motifs and functional regions have been shown to participate in the most critical steps for the correct recognition and incorporation of ribonucleotides [1,3,5,10,11] and are described below (Table 1).

**Table 1:** Location, function and presence in the RNA viral groups of the distinct structural motifs conserved in monomeric RNA-dependent polymerases.

Motif	Location	Presence	Structure	Function
**A**	Palm	RNA-dependent polymerases	β-strand—Helix/loop	Phosphodiester bond formation
				Substrate discrimination
				Interaction with the phosphate moiety of the incoming NTP
**B**	Palm	RNA-dependent polymerases	Loop—Helix	Template binding
				Substrate discrimination
				Hinge for conformational changes
**C**	Palm	RNA-dependent polymerases	β-strand—turn- β-strand	Phosphodiester bond formation
**D**	Palm	RNA-dependent polymerases	Helix—Loop	Structural scaffold
				Protonation of the pyrophosphate leaving group
				Hinge for conformational changes
**E**	Palm	RNA-dependent polymerases	β hairpin	Positioning of the primer 3' OH
**F**	Fingers	RNA-dependent polymerases	Loop- β-strand	Interaction with the phosphate moiety of the incoming NTP
**G**	Fingers	ss(+)RNA viruses, dsRNA viruses	Loop	Part of the template entrance tunnel
**H**	Thumb	ss(+)RNA viruses, dsRNA viruses and RT	Helix—turn—helix	Not yet described
**G**	PA subunit	segmented ss(-) RNA viruses	Helix	Interaction with the priming NTP
**H**	Fingers	segmented ss(-) RNA viruses	β-strand	Stabilization of motif B

ss(+)RNA viruses- single-stranded positive RNA viruses; dsRNA viruses—double-stranded RNA viruses; RT—reverse transcribing viruses; ss(-)RNA viruses—single-stranded negative RNA viruses; NTP—nucleotide triphosphate.

### The structural motifs of monomeric viral RNA-dependent RNA polymerases and reverse transcriptases

#### Motif A

Motif A is located within the palm subdomain and is formed by a β-strand followed by a helical structure or a loop that continues to the fingers subdomain. At the C-terminus of the β-strand, this motif contains one of the invariant catalytic aspartic acid residues present in DNA- and RNA polymerases (Fig 1E).

Single-stranded positive RNA viruses have a characteristic—DX_4_D- conserved sequence, in which the first aspartate corresponds to the catalytic residue. Structural and thermodynamic evidence shows that the second aspartate in motif A plays a key role in the discrimination of NTP over dNTP by forming a hydrogen bond with the ribose 2’ OH moiety [16]. The polymerases of ss(-)RNA viruses and reverse transcriptases lack the C-terminal aspartic acid. The polymerase structures of influenza A and B and the Lacrosse viruses have one conserved lysine, three amino acids downstream of the catalytic aspartic acid, which has been shown to be part of the NTP entrance tunnel [17].

Instead of the second aspartate of motif A, reverse transcriptases have an amino acid with a bulky side chain such as phenylalanine, which is known as “the steric gate” that helps to discriminate between deoxyribonucleotides and ribonucleotides, avoiding the incorporation of the latter by the steric interference of the side chain of this residue with the 2’ OH group of the ribonucleotide [18,19]. Apart from the pair of conserved residues within motif A, residues with aromatic rings four amino acids downstream of the catalytic aspartate are conserved in both positive- and negative single-stranded RNA viruses, which suggest that they play an important role either in the structural stability of the protein due to the hydrophobic nature of the side chains, or in nucleotide binding due to their position within the active site.

#### Motif B

Motif B is located in the transition between the fingers and the palm subdomains. Its structure consists of a loop that connects a β-strand of the fingers and the N-terminal helix of the palm subdomain. The residues of this motif have been shown to participate in binding the template and the incoming nucleotide [20]. The N-terminal loop of motif B is a dynamic structure that participates in template binding. Within this loop, right before the start of the α-helix, both RdRps and RTs have a strictly conserved glycine preceded by a serine in both ss(+) and dsRNA viruses, and a glutamine in retroviruses [10,21]. In the case of influenza A, this glycine is located in a methionine-rich region, but in the arena- and bunyaviruses it is preceded by a glutamine [15,17]. This conserved glycine has been shown to serve as a pinpoint for the loop to change its conformations [20], and mutating this residue results in the complete abolishment of the polymerase function [22].

Single-stranded positive- and dsRNA viruses have a conserved threonine within the α-helix that is located in its N-terminal part and faces the active site of the polymerase. In the next helix turn, also facing the active site, ss(+)RNA viruses have a conserved asparagine, which has been shown to aid in the rNTP selection by correctly positioning the catalytic aspartate in motif A [20]. The structures of segmented ss(-)RNA viruses have two dyads of conserved residues apart from the previously mentioned glycine. One amino acid after this glycine, a phenylalanine and an asparagine are conserved. As mentioned above, this last residue is conserved in ss(+)RNA viruses. Two amino acids downstream there are two conserved hydroxylic residues, either serine or threonine [15,17,23].

#### Motif C

Motif C follows motif B in the palm subdomain, and is formed by a β-strand-loop-β-strand structure. The second catalytic aspartic acid residue conserved in both DNA- and RNA polymerases that coordinates the interactions with the metal ions is located within the loop. Motif C is one of the most conserved regions in viral RNA polymerases; all the loops of ss(+), segmented ss(-), ds- and reverse transcribing RNA viruses have two aspartates preceded by a glycine in ss(+) and dsRNA viruses, a serine in segmented ss(-)RNA viruses, and by a methionine in RT viruses. Both aspartates coordinate the interactions with the metal ions [3]. Previous work in other viral polymerases has shown that any mutation of the first aspartic residue results in a complete loss of RNA polymerase activity, whereas mutations of the second aspartate diminish the polymerase activity or modify the metal cofactor requirements, but do not inactivate the enzyme [24,25]. Viral families such as the dsRNA Birnaviridae, that infects animals, or the ss(-)RNA Mononegavirales order, have an asparagine instead of the second aspartic acid in Motif C. It has been shown that mutating this residue reduces the enzyme’s activity [14]. Moreover, this substitution enables viral RNA polymerases to use manganese instead of magnesium as cofactor [14]. When the asparagine is replaced by an aspartate, the mutated enzyme replicates the viral RNA more efficiently than the wild type. It was suggested that this less efficient polymerase slows the replication kinetics of birnaviruses, which could favor the virus spread [14].

#### Motif D

Motif D follows motif C within the palm subdomain. It is formed by an α-helix and a flexible loop adjacent to the palm’s β-sheet. It is a highly dynamic structure that changes its conformation when the correct nucleotide is bound. It also serves as a structural scaffold for the palm domain, and has been involved in the protonation of the pyrophosphate leaving group after the nucleotidyl transfer reaction [26,27]. Located at the N-terminal end of motif D’s loop, there is one glycine, which has been shown to be conserved in many RdRps from ss(+), ss(-), dsRNA viruses and retroviruses [15,17,21]. It has been argued that this conserved glycine serves as a hinge for this structure that might play a key role in its conformational changes [21].

The second widely conserved residue in motif D is a lysine. It has been proposed that this amino acid, by acting as the general acid that deprotonates the pyrophosphate leaving group, contributes to the rate of nucleotide addition [28]. It is also part of the NTP entrance tunnel in ss(-)RNA viruses [15,17]. Even though this lysine is located in a similar position in the available RdRps crystal structures, it must be underlined that the distance in the primary structure between this conserved residue and the conserved glycine varies in the different viral families.

#### Motif E

Motif E is a unique structural feature of RdRp and RTs, which has been called “the primer grip”. It is a β-hairpin that is located facing the palm subdomain’s β-sheet at the junction with the thumb subdomain. As its name implies, this motif has been shown to act in the correct positioning of the 3’ OH end of the primer [29].

The level of primary sequence conservation around motif E seems to be lower in viral RdRps, despite the fact that all the crystal structures exhibit the characteristic β-hairpin located in the same position [10,21]. One of the most conserved features in viral RNA polymerases is the presence of an aromatic amino acid at the N-terminal moiety of the loop facing motif C. Single-stranded positive RNA viruses of the families Picornaviridae and Caliciviridae, as well as the dsRNA viruses of the family Reoviridae, exhibit a similar arrangement. The former have a leucine and a basic residue, either lysine or arginine, after the aromatic amino acid, while the latter have a glycine and a lysine. There is evidence that basic residues located in the loop of the β-hairpin of Picornaviridae interact with the primer [30]. In the case of the Flaviviridae, the aromatic amino acid is followed by a cysteine and a serine, while in the Orthomyxoviridae and the Bunyaviridae, it is followed by either threonine or valine and a serine. In the Qβ phage replicase the aromatic amino acid is absent, since in this case a serine is followed by a cysteine and a glycine.

Viruses with a protein-priming mechanism such as the Picornaviridae have larger template-binding channels, in which two basic residues with long side chains protruding towards the active site in motif E easily fit [31], while viruses that have *de novo* initiation such as the Flaviviridae [32,33], the bacteriophage ϕ6 [34], and ss(-)RNA viruses [3,17,23,35,36] have more elaborate thumb subdomains and a structure which has been named “the priming loop”, which is a β-hairpin that protrudes towards the active site creating a platform for priming and reducing the space for large side chains in motif E.

At the C-terminus of the β-hairpin, ss(-)RNA viruses have one conserved glycine. Hass et al. [37] have shown that this conserved residue is required by ss(-)RNA viruses polymerases for transcription, but not for genome replication. The position of this glycine in the three-dimensional structures suggests that it might work as a hinge for the thumb domain to move [15,17,23].

#### Motif F

Besides the conserved structural motifs A to E, which practically define the palm subdomain, one additional conserved motif named motif F has been identified in the fingers subdomain of all the crystallized RNA viral polymerases.

This motif extends from the fingers subdomain towards the thumb subdomain as part of the fingertips, directly on top of the palm subdomain active site. This long structure has numerous basic residues that interact with the negatively charged phosphate backbone of the incoming nucleotide, and has been shown to be part of the NTP entrance tunnel in ss(-)RNA viruses [3,32,38]. The only conserved residue in all the motif F structures is an arginine located near its C-terminus.

#### Single-stranded positive and double-stranded RNA viruses polymerase motif G

Gorbalenya et al. [13] and Pan et al. [14] identified the so-called motif G, which is an additional conserved structural motif found in many ss(+) and dsRNA viruses, which is found in the fingers subdomain approximately 120 amino acids upstream of motif A’s catalytic aspartic acid. The consensus sequence of the motif is S-X-G, and forms a loop that is part of the template entrance tunnel.

#### Single-stranded positive, double-stranded RNA polymerases and reverse transcriptases motif H

Cerny et al [10] recently proposed the presence of an additional conserved structural motif located in the thumb subdomain of ss(+), dsRNA, and RT viruses, which they named motif H. It is formed by an helix-turn-helix structure, but there is not a single strictly conserved amino acid within the motif. This motif has been identified based solely on multiple sequence alignments, and its actual function has not been described [10].

#### Segmented single-stranded negative RNA viruses structural motifs G and H

Apart from the conserved structural motifs A-F, Gerlach et al. [15] identified two additional motifs G and H in the segmented ss(-)RNA viral polymerases. These motifs are located in different positions and have functions different from those of the previously named motifs G and H of ss(+) and dsRNA viruses [10,21]. Gerlach’s motif G is found in the C-terminal region of the influenza virus PA subunit, and in the N-terminal half of the LaCrosse virus L protein, facing the active site, and has the sequence RKLL and RYMI, respectively. It has been proposed that the conserved arginine could interact with the priming NTP. The proposed motif H is found, sequence-wise, in the region between motifs A and B, and is located in the fingers subdomain. “on top” of motif B. It has one conserved lysine which has been proposed to stabilize motif B [15].

#### Single-stranded positive RNA viral conserved functional regions

The recently proposed functional regions seem to be conserved in RdRps of ss(+) RNA viruses [2]. Two of them are located in the fingers subdomain and interact with the template RNA strand, while the third functional region is located in the thumb subdomain and binds the nascent RNA strand [2].

Given the availability of crystal structures of polymerases from the four major groups of RNA viruses, i.e., ss(+), ss(-), ds, and reverse-transcribing, we present in this paper a phylogenetic tree built based on comparisons of RdRps and RTs’ tertiary structures, which might help us understand the evolutionary relationships among these enzymes. Prompted by the lack of a crystal of the Ebola virus (EBOV) [39] polymerase, we have used this data to build a homology-based three-dimensional model of the EBOV RdRp domain and, by extension, of all the other viruses that belong to the Mononegavirales order, which include viruses associated with important human pathologies such as measles, rabies, human respiratory syncytial virus, and the Ebola hemorrhagic fever, among many others. The recent availability of ss(-)RNA viral polymerases tertiary structures [15,17,23,40] allowed an evaluation of our approach. As expected, our results demonstrate that the EBOV RdRp shares a homologous catalytic palm subdomain and other functionally important motifs with the other viral RdRp described thus far. As argued here, the evolutionary conservation of three-dimensional features common to all the monomeric polymerases analyzed here help explain the recent reports of the successful use against the EBOV infection of antivirals originally targeted to inhibit other types of polymerases, such as RTs and DNA-dependent DNA polymerases.

## Materials and Methods

### Structural comparisons and dendogram construction

The RdRp crystal structures employed in this study include: human rhinovirus polymerase (PDB:1XR7); poliovirus (PDB:3OL6); human enterovirus 71 (PDB:3N6L); foot-and-mouth disease virus (PDB:1U09); encephalomyocarditis virus (PDB:4NZ0); Norwalk virus (PDB:3BSO); rabbit hemorrhagic disease virus (PDB:1KHV); Sapporo virus (PDB:2CKW); Japanese encephalitis virus (PDB:4HDH); dengue virus (PDB:2J7U); bovine viral diarrhea virus (PDB:1S48); hepatitis C virus (PDB:1C2P); Qβ-phage (PDB:3MMP); infectious pancreatic necrosis virus (PDB:2YI9); infectious bursal disease virus (PDB:2PUS); φ6 phage (PDB:1HHS); reovirus (PDB:1MUK); simian rotavirus (PDB:2R7Q); human immunodeficiency virus type 1 (PDB:3DLK); moloney murine leukemia virus (PDB:4MH8); human immunodeficiency virus type 2 (PDB:1MU2); *Tribolium castaneum* telomerase (PDB:3KYL); bat influenza A virus (PDB:4WSB); human influenza B virus (PDB:4WRT); LaCrosse virus (PDB:5AMQ).

Pairwise structural comparisons between the different RdRps and RTs mentioned above were performed with the Secondary Structure Matching (SSM) program [41] included in the PDBe web server. In the case of reverse transcriptases, both the connection and the RNase H domains were deleted for the comparisons. All the crystals have a resolution of 3 Å or higher. The results of each set of comparisons allowed the construction of a matrix that included the number of residues in each of the structures, the Root-mean Square Deviation (RMSD), and the number of aligned residues.

A geometric distance measure was then estimated for each of the comparisons using the Structural Alignment Score [42], which is calculated according to the following formula: (RMSD x 100)/number of aligned residues.

The program FITCH, included within the PHYLIP package, was used to transform the geometric distance into an evolutionary distance and FigTree (http://tree.bio.ed.ac.uk/software/figtree/) was used to visualize the resulting tree.

### Ebola virus L protein study: remote homology detection and three-dimensional structure modeling

The search for homologs of the EBOV polymerase and the three-dimensional structure modeling of the EBOV RdRp domain were performed using the PHYRE web server version 2.0 [43]. One Zaire Ebola virus L protein sequence (Sierra Leona, Makona-G3686.1; AIE11922) from the current outbreak was downloaded from NCBI’s Viral Genome Resource (http://www.ncbi.nlm.nih.gov/genome/viruses/). The sequence was edited according to the information provided by the Conserved Domain Database [44], leaving only the fragment of the sequence which corresponds to the entry “Mononegavirales RNA dependent RNA polymerase” (CDD 250248). This edited fragment of the protein was used as the PHYRE version 2.0 server query sequence.

The three-dimensional model and its images were edited with Chimera 1.8 [45].

### Mononegavirales L protein secondary structure-based multiple sequence alignment

Secondary structure-based multiple sequence alignments were built using the PROMALS3d web server [46]. For each EBOV species, one L protein sequence was randomly chosen with the exception of the Zaire EBOV, for which two sequences were chosen, one from the 1976 Yambuku-Mayinga outbreak and another one from the current outbreak. The accession codes for the sequences employed here are listed: Reston Ebola virus: NP_690587.1; Sudan Ebola virus: YP_138527; Tai Forest Ebola virus: YP_003815431; Bundibugyo Ebola virus: YP_003815440; Zaire Ebola virus 1976 Yambuku-Mayinga outbreak: NP_06625; Zaire Ebola virus 2014 sample ManoRiver-G3823: AIG96450.

For the Mononegavirales multiple sequence alignment, the different L-protein “Mononegavirales RNA-dependent RNA polymerase” (CDD 250248) domains from the various genera belonging to the Mononegavirales families were chosen. A total of 52 sequences were included for the alignment. These are listed below, and include the Borna disease virus: NP_042024; Lloviu cuevavirus: YP_004928143; Marburg marburgvirus: YP_001531159.1; avian metapneumovirus: YP_443845; avian paramyxovirus 6: NP_150063; Beilong virus: YP_512254; bovine parainfluenza virus 3: NP_037646; bovine respiratory syncytial virus: NP_048058; canine distemper virus: NP_047207; dolphin morbillivirus:NP_945030; fer-de-lance virus:NP_899661; human metapneumovirus: YP_012613; human parainfluenza virus 1:NP_604442; human parainfluenza virus 2:NP_598406; human parainfluenza virus 3:NP_067153; human respiratory syncytial virus:NP_056866; Mapuera virus:YP_001249278; measles virus:NP_056924; mumps virus:NP_054714; Newcastle disease virus B1:NP_071471; parainfluenza virus 5:YP_138518; peste-des-petits-ruminants virus:YP_133828; pneumonia virus of mice J3666:YP_173335; porcine rubulavirus:YP_001331035; rinderpest virus: YP_087126; Sendai virus: NP_056879; simian virus 41:YP_138510; Australian bat lyssavirus:NP_478343; bovine ephemeral fever virus:NP_065409; European bat lyssavirus 1:YP_001285392; Hirame rhabdovirus: NP_919035; infectious hematopoietic necrosis virus:NP_042681; lettuce yellow mottle virus: YP_002308376; maize Iranian mosaic virus:YP_002308459; maize fine streak virus: YP_052849; Mokola virus:YP_142354; northern cereal mosaic virus:NP_597914; potato yellow dwarf virus:YP_004927971; rabies virus:NP_056797; rice yellow stunt virus:NP_620502; snakehead virus:NP_050585; sonchus yellow net virus:NP_042286; spring viraemia of carp virus:NP_116748; taro vein chlorosis virus:YP_224083; vesicular stomatitis Indiana virus:NP_041716; viral hemorrhagic septicemia virus Fil3:NP_049550.

## Results and Discussion

### Tertiary structure-based phylogeny of RdRps and RTs

RNA-dependent RNA polymerases have been used as evolutionary markers because of their presence in all RNA viruses and their several conserved regions and number of amino acids [10,38,47–49]. However, the high level of primary structure divergence among the different groups of RNA viruses has hindered their usefulness as a tool for obtaining insights into deep phylogenies and the evolutionary relationships between the different viral families.

**Fig 2:**
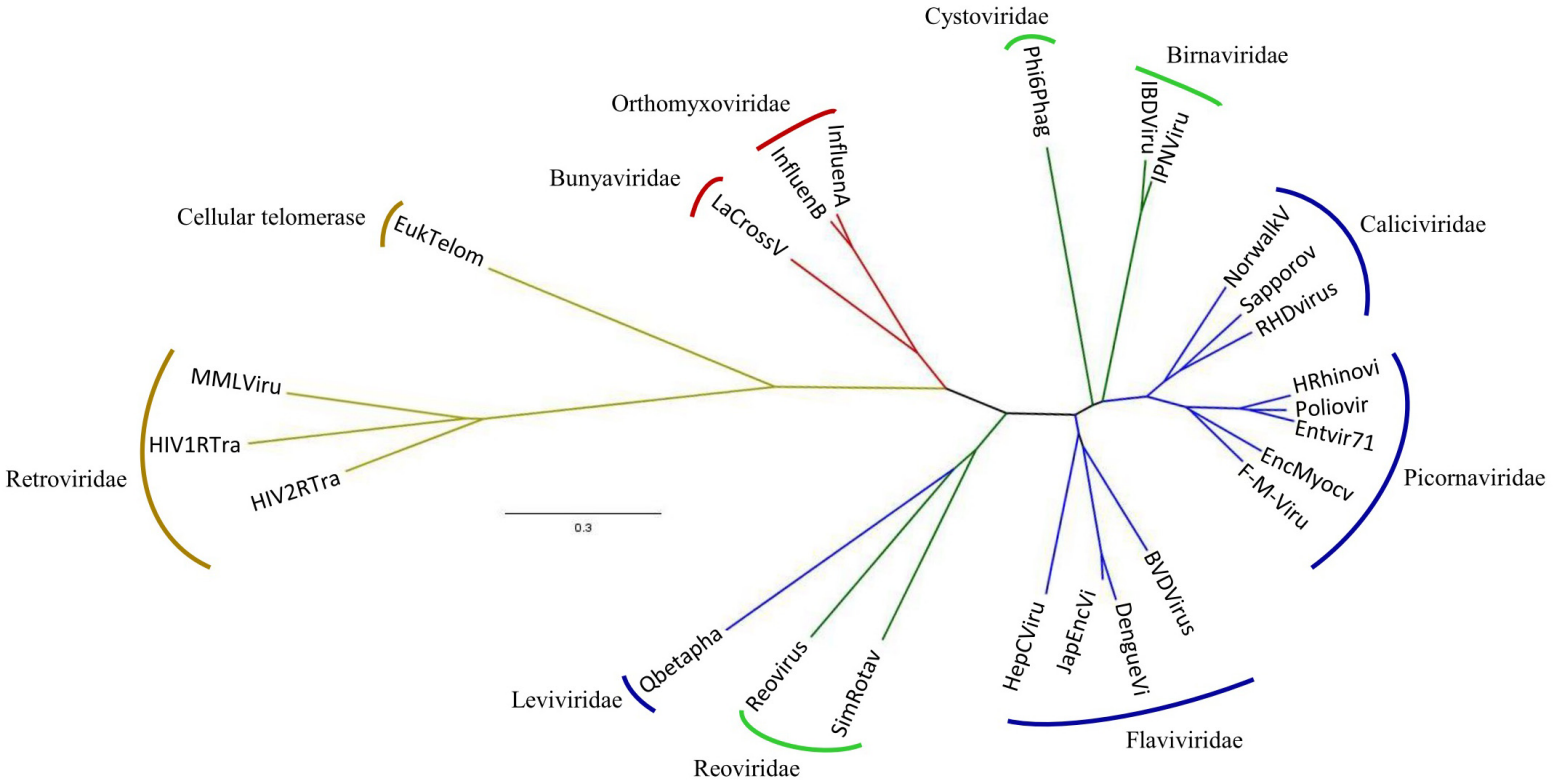
Dendogram based on the structural comparisons of RNA-dependent polymerases. The colors of the branches stand for: red, single-stranded negative RNA viruses; blue, single-stranded positive RNA viruses; green, double-stranded RNA viruses; yellow, retrotranscribing viruses and eukaryotic cell telomerase. The names of the viral families are outside each clade. The tertiary structures of the polymerases used for the analysis were: Hrhinovi-human rhinovirus, PDB:1XR7; Poliovir-poliovirus, PDB:3OL6; EntVir71-human enterovirus 71, PDB:3N6L; F-M-Viru—foot-and-mouth disease virus, PDB:1U09; EncMyocv-encephalomyocarditis virus, PDB:4NZ0; Norwalkv-Norwalk virus, PDB:3BSO; RHDVirus-rabbit hemorrhagic disease virus, PDB:1KHV; Sapporov-Sapporo virus, PDB:2CKW; JapEncVi-Japanese encephalitis virus, PDB:4HDH; DengueVi-Dengue virus, PDB:2J7U; BVDVirus-bovine viral diarrhea virus, PDB:1S48; HepCViru-hepatitis C virus, PDB:1C2P; Qbetapha-Qβ-phage, PDB:3MMP; IPNVirus-infectious pancreatic necrosis virus, PDB:2YI9; IBDVirus-infectious bursal disease virus, PDB:2PUS; Phi6phag-φ6 phage, PDB:1HHS; Reovirus-reovirus, PDB:1MUK; SimRotav-simian rotavirus, PDB:2R7Q; HIV1RTra-human immunodeficiency virus type 1, PDB:3DLK; MMLVirus-Moloney murine leukemia virus, PDB:4MH8; HIV2RTra-human immunodeficiency virus type 2, PDB:1MU2; EukTelom-*Tribolium castaneum* telomerase, PDB:3KYL; InfluenA—bat influenza A virus, PDB:4WSB; InfluenB-human influenza B virus, PDB:4WRT; LaCrossV-LaCrosse virus (PDB: 5AMQ).

A relatively recent important alternative to the construction of primary structure-based phylogenies are evolutionary trees based on the comparison of tertiary structures. This method has proven to be particularly useful when trying to assess the evolutionary relationship between homologous proteins with high levels of sequence divergence [50]. The unrooted phylogeny we have constructed using this approach is shown in Fig 2 and is part of this trend. It exhibits several different well-defined branches, each of them clustering one or two viral families. One of the branches groups the ss(+)RNA viruses of the families Picornaviridae and Caliciviridae. Double-stranded RNA viruses are not grouped into a single branch. There are two branches with only dsRNA viruses close from one another in the tree. One of which corresponds to the Birnaviridae family, and the other to the Cystoviridae family. The fact that they are not grouped in one single clade might be due to the presence of a circular permutation in the Birnaviridae polymerase that alters the topology of the palm subdomain. A major clade groups dsRNA viruses of the family Reoviridae and ss(+) viruses of the family Leviviridae, with this latter diverging from the dsRNA branch. Another one clusters the ss(+)RNA viruses of the family Flaviviridae. In the tree shown in Fig 2, one branch groups ss(-)RNA viruses, i.e., the LaCrosse virus and the Orthomyxoviridae family polymerases. The VSV L protein structure is not included in this tree because its resolution is below the 3A threshold. As shown in Fig 2, the longest and most distant branch groups together the RTs with the eukaryotic telomerase stemming close to the root of this clade.

Phylogenetic trees of ss(-)RNA viruses using the most conserved regions of the RdRp [38,49] show that segmented negative-stranded RNA viruses are phylogenetically related and are grouped in a separate clade from the Mononegavirales order. The latter are a monophyletic group, and each family (Paramyxoviridae, Rhabdoviridae and Bornaviridae) has its own branch, with the exception of the family Filoviridae, which stems from the Pneumovirinae subfamily within the Paramyxoviridae node.

The monophyly of ss(-)RNA viral polymerases is supported by additional biochemical and structural data. Transcription and replication of all ss(-)RNA viruses is similar. First the mRNA, which is modified in a later stage, is synthesized using the genomic RNA as template. A complementary positive strand is also formed, which is used as the template for the synthesis of the genomic negative sense RNA, which is then packed inside the virions. Other shared features of negative-strand RNA viruses are the fact that the polymerase template consists of a ribonucleoprotein complex in which the viral nucleoprotein is bound to the genomic RNA [4] and, that the polymerases use a *de novo* initiation mechanism [15,17,23,35,36]

The mRNA of ss(-)RNA viruses must be capped to be recognized by the cellular protein synthesis machinery. Non-segmented RNA viruses have within the L protein a capping domain located bordering one of the faces of the RdRp domain forming the template channel. No structural homologs are known for this domain, and the capping mechanism is different from that of eukaryotic cells and segmented ss(-)RNA viruses. It works by the attack of a guanosine nucleotide to a histidine residue covalently bound to the 5’ end of the RNA, i.e., it is said to have GDP polyribonucleotidyl transferase activity [40]. On the other hand, segmented RNA viruses carry a protein, which can be part of the polymerase protein (Arenaviridae and Bunyaviridae) or can be synthesized as a multi-domain protein (Orthomyxoviridae), that “steals” the capping from the cellular proteins, which are then used as transcription primers. This process has been named cap-snatching [51,52].

The ss(-)RNA viral polymerases are big complexes endowed with many different functional domains. In the case of the Orthomyxoviridae family, the polymerase is a heterotrimeric complex formed by the proteins PA, PB1 and PB2. The PB1 protein contains the RdRp domain [17,23]. In the case of the LaCrosse orthobunyavirus [15], the polymerase complex is part of the L protein, a 2250 amino acid-long protein that includes at least three distinct functional domains: endonuclease, PA-C like, and RdRp domains [15]. Despite the different coding strategies and a lack of primary sequence homology, structural comparisons between the Orthomyxoviridae and the Bunyaviridae polymerases show a linear correlation between the complexes and homologous functional domains, i.e. PA and the N-terminus of the L protein; PB1 and the central region of the L protein; and PB2 and the C-terminus of the L protein.

Electron microscopy (EM) studies of the VSV polymerase have revealed certain differences in the overall structure and organization of non-segmented ss(-)RNA viruses [40] in comparison with the polymerases from segmented ss(-) RNA viral polymerases [15,17,23]. The Mononegavirales L protein consists of five domains, i.e., the RdRp, capping, connector, methyltransferase and the C-terminal domains. It has a number “6” shape, in which the bottom part is formed by the RdRp and the capping domain, and the top is formed by the connector, methyltransferase and C-terminal domains [40].

The different location of the capping enzymes (N-terminal in segmented ss(-)RNA viruses vs C-terminal in the Mononegavirales), the differences in the quaternary structure of the polymerases, and the distinct capping mechanisms indicate a ss(-) RdRp ancestor and later accretion events during which the complementary functional domains were independently acquired.

Different attempts to construct evolutionary trees of viral RdRps and RTs based on structural comparisons including this work [10,53] yield similar but not identical results. This can be easily understood as the outcome of the different methodological approaches developed by Cerny et al. [10], Mönttinen et al [53], and ourselves. Cerny et al. [10] used a dual method. First they made structural multiple alignments to improve a primary sequence-based alignment, followed by the construction of a matrix with the structural “phenotypic” features of viral RdRps and RTs, and then they combined the two results to construct an unrooted evolutionary tree. On the other hand, Mönttinen et al. [53] made automated comparisons of the available DNA- and RNA polymerases structures in order to construct a normalized geometrical distance matrix, which was in turn converted to an “evolutionary” distance matrix, which was then used to build a phylogenetic tree. For this last step, both Mönttinen et al. [53] and our group (Fig 2), used the FITCH algorithm, since other algorithms such as KITSCH assume that all the species included in the analysis are contemporary and that there is a molecular clock.

While the results of Cerny et al. [10], Mönttinen et al. [53] and our group share many similarities and exhibit some differences, it is equally significant that none of them is consistent with the Baltimore classification of RNA viruses [54]. In all three reports, polymerases from the ss(+) and dsRNA viruses are interspersed, and in the trees of Mönttinen et al. [53] and ours (Fig 2), there is one branch that includes polymerases of both Leviviridae and Reoviridae. The Qβ-phage, a ss(+)RNA virus of the family Leviviridae, is distant from all other ss(+)RNA viruses in the three phylogenies, forming one independent branch in the tree of Cerny et al. [10], and diverging from the Reoviridae family in the other two trees (Fig 2) [53]. In all three cases, the most distant and longest branch corresponds to reverse transcriptases, both viral and cellular. The recent availability of ss(-)RNA viral polymerases [15,17,23,40] allowed us to include them in the phylogenetic tree (Fig 2). Although the structure of the mononegaviral VSV polymerase has been recently reported [40], we have not included it in our tree due to its low resolution (3.8A).

### A theoretical 3D structure of the Ebola virus RNA polymerase: a model for non-segmented single-stranded RNA viral polymerases

Due to the biomedical relevance of ss(-)RNA viruses and the lack of structural information of their RdRp, several attempts had been made to model the polymerase domain based on the homology with other RNA viruses whose polymerases had already been crystallized. A predicted model of an Arenavirus RdRp domain [38] built using the hepatitis C virus polymerase as a reference showed a remarkably similar structure to the rest of the RdRps and included the entire palm subdomain, fragments of the fingers subdomain, and the structural elements inserted between the palm, as well as the N-terminal region of the thumb subdomain. Later works by Hass et al. [37] demonstrated that the predicted structural model of the Arenavirus polymerase was correct, and that several of the conserved residues located within the conserved structural motifs A-F are relevant for the proper function of the enzyme, and that mutations to most of these residues completely abolish the polymerase’s catalytic reaction.

The current epidemic of the EBOV hemorrhagic fever is by far the biggest outbreak of this disease since its discovery in 1976. The high mortality rates ranging from 40 to almost 90% [55–57], combined with the lack of approved vaccines and effective treatments against the virus, have pushed the biomedical community in the search towards a better understanding of this pathogen.

EBOV is part of the family Filoviridae which, together with the families Rhabdoviridae, Paramyxoviridae and Bornaviridae, forms the Mononegavirales order. The members of this highly diverse group of viruses all have a linear, monopartite, negative strand RNA genome, share transcription and replication strategies, and exhibit a conserved arrangement of at least five genes that encode for nucleoprotein, phosphoprotein (VP35 in EBOV), matrix protein, glycoprotein and L protein [58]. The filoviruses have two additional proteins located between the glycoprotein and the L-protein, VP30, which is an essential cofactor for the Filoviridae mRNA synthesis [59], and VP24, which participates in nucleocapsid formation [60], viral assembly and budding [61] as well as in viral evasion from the host immune system [62].

The L protein of Ebola virus is a multifunctional protein about 2210 amino acids long with a molecular weight of approximately 250 kDa engaged in viral transcription, genome replication, mRNA capping, mRNA methylation and polyadenylation [63,64]. Poch et al [65] identified six blocks with a high degree of conservation in the entire L protein of the five species of Mononegavirales available 25 years ago, and concluded that each block could be performing a particular function of the L protein. The work of Liang et al. [40], has shown that conserved blocks I-III are located in the RdRp domain; blocks IV and V are found within the capping domain; and conserved block VI is part of the methyltransferase domain.

The description of the VSV L protein structure to a 3.8 A resolution [40] has provided new insights on the overall three-dimensional arrangement of the Mononegavirales polymerase (vide supra), including several conserved traits with the previously characterized RdRps and RTs. Prompted by the lack of a tertiary structure of the EBOV polymerase to an atomic resolution level, we have developed a three-dimensional model of the RdRp domain of the EBOV L protein by using the PHYRE 2.0 web server and, with the addition of a Mononegavirales L protein secondary structure-based multiple sequence alignment, identify conserved residues within the enzyme that might help in the design of specific drugs that could counteract the EBOV epidemic.

The best match yielded by the PHYRE 2.0 server to our EBOV polymerase sequence corresponds to the bat influenza A virus (PDB code 4WSB, chain B) [17]. Only 253 residues of the EBOV polymerase could be aligned with confidence levels of 91.7%, and the identity between the fragments of the two proteins is of 12% (S1 Fig). Although the ss(-)RNA LaCrosse virus polymerase is now available [15], its use as a template in the alignment and model prediction were not encouraging. The sequence coverage is larger compared with other alignments (358 residues aligned) and the identity was 13%, but the predicted model only ranked 15^th^ with a confidence level of 33.9%.

**Fig 3:**
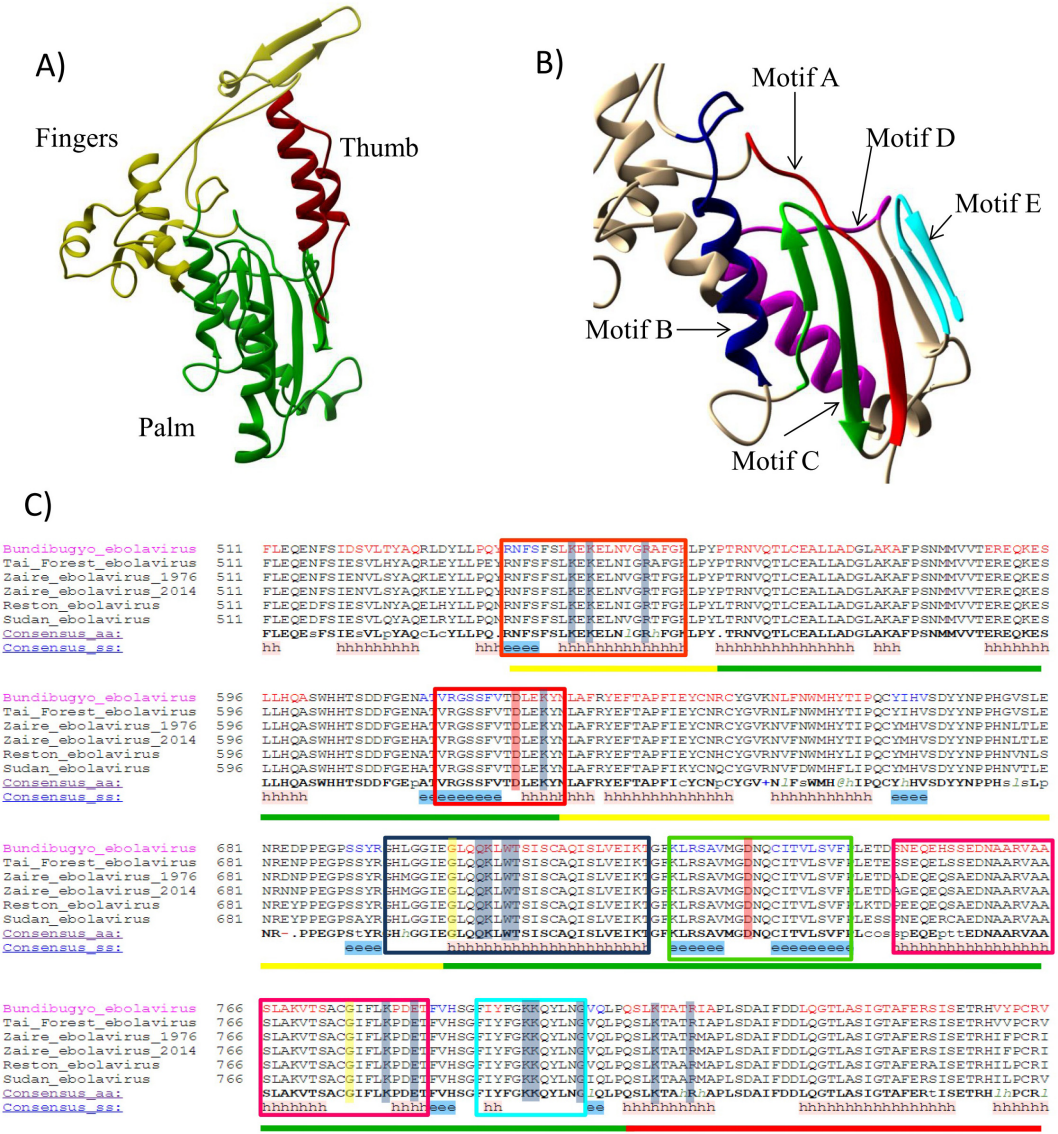
Predicted model of the EBOV polymerase. (A) Only the residues that could be aligned with a 90% confidence or higher are drawn. The color code is the same as in Fig 1; (B) Conserved structural motifs in the Ebola virus polymerase. The motifs are colored as in Fig 1: motif A, red; motif B, dark blue; motif C, green; motif D, magenta; motif E, cyan. The image has been amplified for a better view of the active site; (C) Secondary structure prediction of the Ebola virus RNA-dependent RNA polymerase. Only the fragment that could be confidently aligned according to the PHYRE results is shown. The color lines under the sequence match the three structural subdomains and are the same as Fig 3A). The color frames surrounding the sequence match the conserved structural motifs depicted in Fig 1). Residues involved in metal ion coordination are highlighted in red; conserved residues involved in template-primer interactions are highlighted in blue; conserved residues likely participating in structural stability and motion are highlighted in yellow.

Our predicted three-dimensional model of the EBOV polymerase allowed the identification of the fingers-, palm-, and thumb subdomains structures and in the same sequential order of DNA- and RNA polymerases, i.e., fingers-palm-fingers-palm-thumb. This is consistent with the recently reported structure of the VSV L protein [40]. The fingers include residues 417–439 and 489–563, the palm subdomain is formed by residues 440–488 and 563–666, and the thumb subdomain includes residues 667–704 (Fig 3A). Adding the secondary structure-based multiple sequence alignment, we were able to identify motifs A to F in our model, which are six of the conserved motifs in the RdRps and in the RTs crystallized so far [1,10,66]. We could not identify the recently proposed motifs G and H of segmented ss(-) RNA viruses [15], which are not part of the active site. The fragment of our predicted three-dimensional EBOV polymerase model is in excellent agreement with the recently published structure of the VSV L protein [40], in which the conserved palm subdomain with structural motifs A to F are observed.

The EBOV polymerase model presented here shows that motif A is formed by a β-strand followed by a ten amino acids-long loop (Fig 3B). The aspartic acid 483 of the EBOV L protein’s motif A is conserved in all the Mononegavirales. This residue matches the catalytic amino acid and is located in the same position as in all the other viral RNA polymerases. It is followed by leucine, glutamate, lysine, tyrosine and asparagine. The lysine residue is conserved in both segmented and non-segmented ss(-)RNA viruses, and is part of the NTP entrance tunnel [17]. In spite of its high level of conservation, Hass et al. [37] proved that the polymerase maintained a normal level of proficiency when this residue was substituted with alanine. It is followed by a residue with an aromatic ring (S2 Fig), which is conserved in both ss(+) and ss(-)RNA viral polymerases. Nine amino acids after the catalytic aspartate, there is one strictly conserved arginine in the Mononegavirales order (S2 Fig). This residue is located in the fingers subdomain, relatively far from the active site, and might be involved in interactions with some of the other functional domains of the L protein, or with the proteins required for the transcription and replication processes. Motif A is found nested within Poch et al. [65] conserved block III.

The EBOV polymerase model’s motif B is formed by a loop followed by a long α-helix. The sequence of the loop and the N-terminus of the helix are GGIEGLQQKLWT. According to its position in the model, the third glycine corresponds to the conserved glycine of RdRps and RTs (vide supra). The position in our model of glutamine 564, lysine 565, tryptophan 567 and threonine 568 suggest that they might be involved in the interactions with the incoming nucleotide. These five residues are highly conserved in the Mononegavirales order with a few exceptions (S2 Fig). Their high level of conservation suggests that their interactions are required for the proper functioning of the enzyme, and their position in the EBOV RNA polymerase three-dimensional structure presented here compared with the known viral RdRps crystal structures hints that these conserved residues could be involved in ribonucleotide selection over dNTPs.

The predicted motif C has the characteristic structure β-strand-loop-β-strand, and its loop has one aspartate residue within the sequence MGDNQ that matches the second strictly conserved amino acid (Asp593) (Fig 3C). The model presented shows that the aspartate and the asparagine are in position to interact with the metal ions and complete the nucleotidyl transfer reaction. The Mononegavirales polymerase sequence has the tetrad GDNQ conserved amongst all its families, with the sole exception of the genus Novirhabdovirus, in which the glutamine has been substituted by a valine (S2 Fig). Directed mutagenesis in viruses belonging to other families of the Mononegavirales, i.e. Rhabdoviridae and Paramyxoviridae, have shown that mutations to the aspartate or the asparagine of Motif C completely abolish the enzymatic activity [67–69]. The strict functional dependence on the asparagine of motif C has to be associated with differences in the active site architecture which will have to be unraveled once the EBOV polymerase crystal is available.

The EBOV polymerase model presented here predicts that motif D is formed by an α-helix followed by a long loop (Fig 3A and 3B). The helical structure has a predominance of hydrophobic residues, which is consistent with its role as a structural scaffold, while the loop is formed by the sequence GIFLKPDET. The Mononegavirales secondary structure-based multiple alignment shows the presence of hydrophobic residues in the helical structure followed by a loop with the consensus sequence G-(L/H/I)-X-(L/I)-K-X2-E-T (S2 Fig).

Glycine (Gly635), located at the N-terminal end of motif D’s loop (Fig 3), corresponds to the glycine that has been shown to be conserved in many RdRps from ss(+), ss(-), dsRNA viruses and retroviruses [15,17,21]. Lysine 639, which is conserved in the Mononegavirales (Fig 3 and S2 Fig), may be the general acid identified in other RdRps, that deprotonates the leaving pyrophosphate group. Finally, glutamic acid 642, which is also highly conserved in the Monegavirales order, (S2 Fig) might be participating in the interactions with the incoming nucleotide.

The EBOV polymerase motif E has the characteristic β-hairpin structure and the sequence FIYFGKKQYL (Fig 3C). In the Mononegavirales its conservation level is low compared with the rest of the motifs, with only the triad of residues (F/Y/M)-(G/S/N)-K exhibiting conservation levels above 60% (S2 Fig). As noted above, motif E has a conserved residue with an aromatic ring, which in the case of the EBOV model could be tyrosine 651 or phenylalanine 652. Although Poch et al. [65] identified within block III a conserved region that was named motif D, the structural alignment and the three-dimensional predicted model presented here shows that it corresponds to structural motif E and the first residues of the thumb subdomain (vide infra) (Fig 3C and S2 Fig).

Our EBOV polymerase model lacks motif F. Nevertheless, analysis of the PHYRE 2.0 alignments with other polymerases such as encephalomyocarditis virus 3dpol and Sapporo virus RdRp and their three-dimensional structures, allowed the identification of a region approximately 70 amino acids upstream of motif A that could correspond to motif F (Fig 3 and S2 Fig). The sequence of this region has four basic residues, FSLKEKELNVGRTFGK. Three of these four basic residues, as well as phenylalanine, are conserved in the Mononegavirales polymerase (S2 Fig). Poch et al [65] identified conserved block II and proposed that, due to the presence of several basic residues, it might be an RNA binding domain. Our work suggests that motif F corresponds to Poch et al. [65] conserved block II.

The last residues of the EBOV polymerase that could be confidently aligned with other polymerases match the N-terminal helices of the thumb subdomain. Two helices were identified. The first one, which is closer to the active site, has two basic residues, one lysine and one arginine (Lys 668 and Arg 672), which are conserved in all the Mononegavirales (S2 Fig), and that could be interacting with the primer strand in the polymerase active site. The second helix, which may have a structural stabilization role, is mainly hydrophobic and exhibits a higher variability without any conserved residue. Even though this predicted region is helical, the connectivity between the predicted helices does not match the motif H proposed by Cerny et al [10].

The EBOV polymerase model presented here lacks the priming loop present in RNA viral polymerases that use a *de novo* initiation mechanism, because the modelling technique we have used only exhibits the enzyme fragment with good confidence levels, and does not include the additional L protein functional domains. This is a strong limitation of our model, since in the ss(-) mononegaviral VSV polymerase structure recently published [40] the priming loop can be seen protruding from the cap domain into the active site.

**Fig 4:**
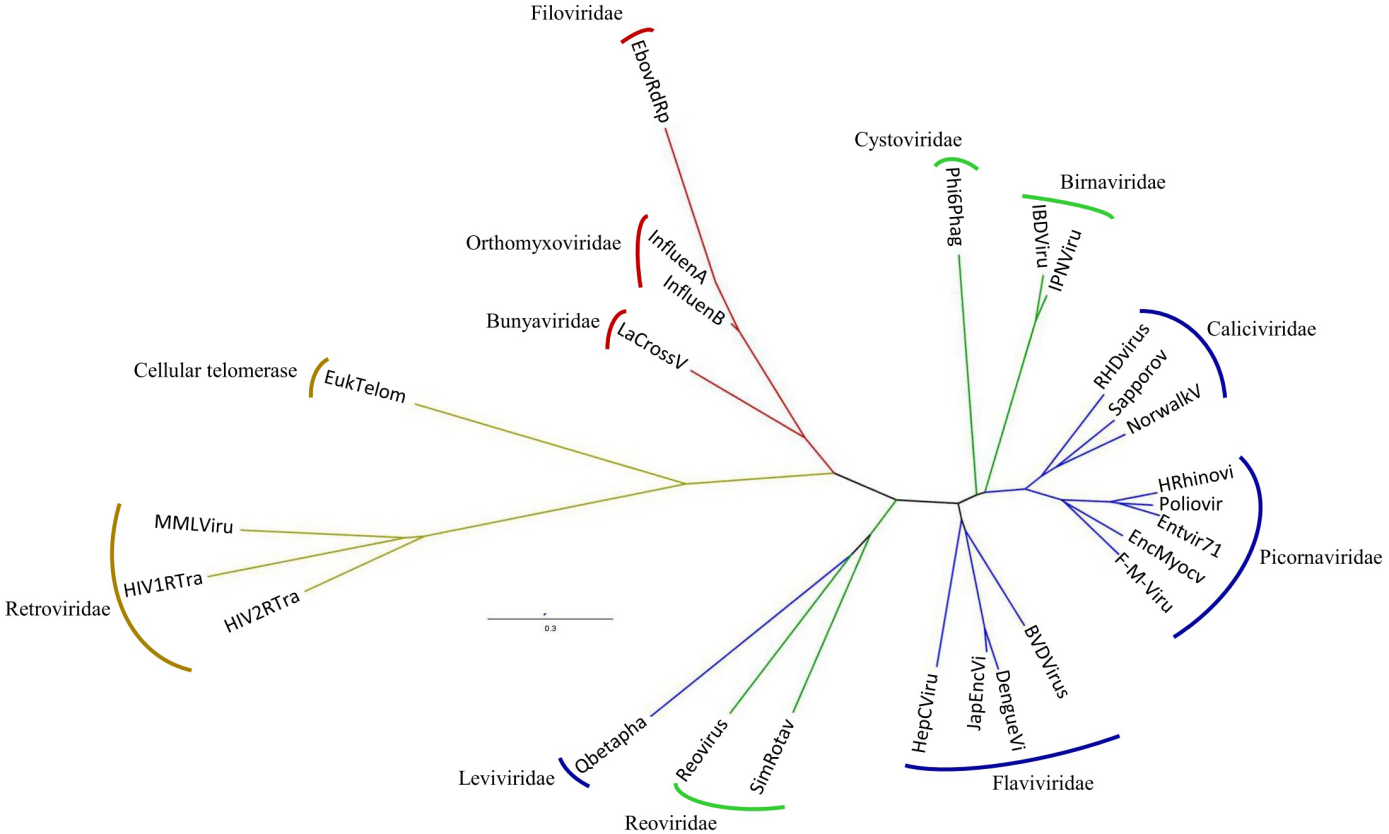
Dendogram based on the structural comparisons of RNA-dependent polymerases including the EBOV RdRp predicted model. Ebola virus predicted model = EbovRdRp. The colors of the branches as well as the crystals used for the dendogram construction are the same as in Fig 2.

We have included our EBOV polymerase model in the RdRps and RTs structure-based phylogenetic tree. As shown in Fig 4, it groups well with the polymerases of ss(-)RNA viruses. S3 Fig shows the high level of structural conservation in the RdRp domains from the recently published single-stranded negative RNA viruses and our EBOV polymerase model. It thus appears that all ss(-)RNA viral polymerases diverged once from a RdRp and RT common ancestor and underwent a later separation that led to the segmented ss(-) and non-segmented ss(-)RNA viral polymerases.

### RNA-dependent RNA polymerases as therapeutic targets

An overwhelming majority of the recent emergent human epidemics are caused by RNA viruses [70], and in spite of the major advancements that have been obtained regarding the treatment of hepatitis C virus [2] and human immunodeficiency virus infections [71], as of today there is no specific drug designed to counteract several of these highly pathogenic diseases [72], including the EBOV infection with its high mortality rate. The fact that RNA-dependent RNA polymerization is an essential process for the viral cycle makes it a very attractive target for the development of antiviral drugs [20]. Indeed, most of the antivirals currently approved are drugs aimed at inhibiting the activity of this crucial enzyme [71], including Brincidofovir (CMX-001), Lamivudine and Favipiravir (T-705), which are being tested against EBOV and have proven to have antiviral activity *in vitro* or *in vivo* [73–75] by inhibiting the polymerase activity. Brincidofovir is a nucleoside phosphonate analog that inhibits DNA chain extension and has proven to be effective against the double-stranded DNA viruses of the families Adenoviridae, Poxviridae and Herpesviridae [76–78], and is already in the late stages of trials as an antiviral for the aforementioned pathogens [79,80].

Lamivudine is a nucleoside-analog reverse-transcriptase inhibitor (NRTI) that also acts as a chain terminator due to its lack of a 3’hydroxyl end [81]. It has been used for many years as an antiretroviral drug in the treatment of hepatitis B chronic infection, although due to the resistance rates it is no longer a first-line drug [82]. It has also been employed against human immunodeficiency virus infections, usually as part of a combination of a multi-drug treatment [83]. During the current EBOV outbreak, it was reported that the treatment with lamivudine early in the infection resulted in the cure of 13 out of 15 patients [84].

Favipiravir is a selective inhibitor of RdRps. Once inside the cell, this nucleotide analog is phosphoribosylated by cellular enzymes and forms favipiravir RTP, which prevents further nucleotide incorporation [85]. This RdRp inhibitor has been shown to have *in vitro* and/or *in vivo* antiviral activity against a wide array of human-infecting RNA viruses, including ss(-) viruses such as influenza virus [86], arenaviruses [87], and bunyaviruses [88]; ss(+)RNA viruses such as flaviviruses [89,90], alphaviruses [91], picornaviruses [85] and noroviruses [92]. It was recently reported that Favipiravir (T-705) has *in vivo* antiviral activity against Zaire EBOV in a mouse model [74].

Nucleotide/nucleoside analogues are drugs aimed at the active site of RNA- and DNA polymerases that compete with the natural substrates for incorporation into the nascent nucleic acid strands, and may act either as chain terminators or as mutagenic agents [93,94]. Protein crystal structures have played a key role in the development of new drugs, since they allow the visualization of the interactions that take place inside and between proteins, which in turn, helps to unravel the atomic interactions that occur between an enzyme and its substrates. They have also been useful in determining which point mutations generate resistance to certain drugs [2,95–97].

No polymerase is endowed with absolute template- or substrate specificity [27,98–100], and the available crystal structures of complexes of DNA- and RNA polymerases with nucleotides or nucleotide analogues all exhibit similar binding mechanisms [30,101–105]. The incoming nucleotide has several interactions with key residues within the active site in order to be correctly positioned for the nucleophilic attack. The triphosphate moiety of the incoming nucleotide interacts with the strictly conserved aspartic acid residues of the palm domain’s motifs A and C which are, in turn, interacting with two divalent metal ions. The coordination of this moiety is completed by interactions with basic residues of the fingers domain’s motif F, which is present in both RdRps and RTs, but absent in DNA-dependent DNA polymerases. Instead, the analogous region in these enzymes is an α-helix, named helix O, which has several basic residues that point towards the active site and coordinate the triphosphate region of the incoming nucleotides [101]. The sugar moiety of the nucleotide interacts with residues in motif A and, in the case of RdRps, with residues in motifs A and B. As mentioned above, residues in motif A play a key role in the discrimination of the correct substrate. DNA-dependent DNA polymerases and RT have a residue with a bulky side chain such as glutamate, tyrosine or phenylalanine that serves as a steric barrier that prevents the incorporation of ribonucleotides into the nucleotide binding pocket [18]. The selection of ribonucleotides in RdRps is determined by the interactions of the 2’ OH moiety with the second conserved aspartate in motif A and the conserved asparagine in motif B of ss(+) and ds RNA viruses. Finally, most of the interactions of the incoming nucleotide base moiety are made with the template and the primer bases. The completion of the abovementioned interactions is a major determinant of the intrinsic fidelity of these enzymes, which is enhanced by the presence of an exonuclease domain in many DNA polymerases, which is clearly a later evolutionary addition [7–9].

**Fig 5:**
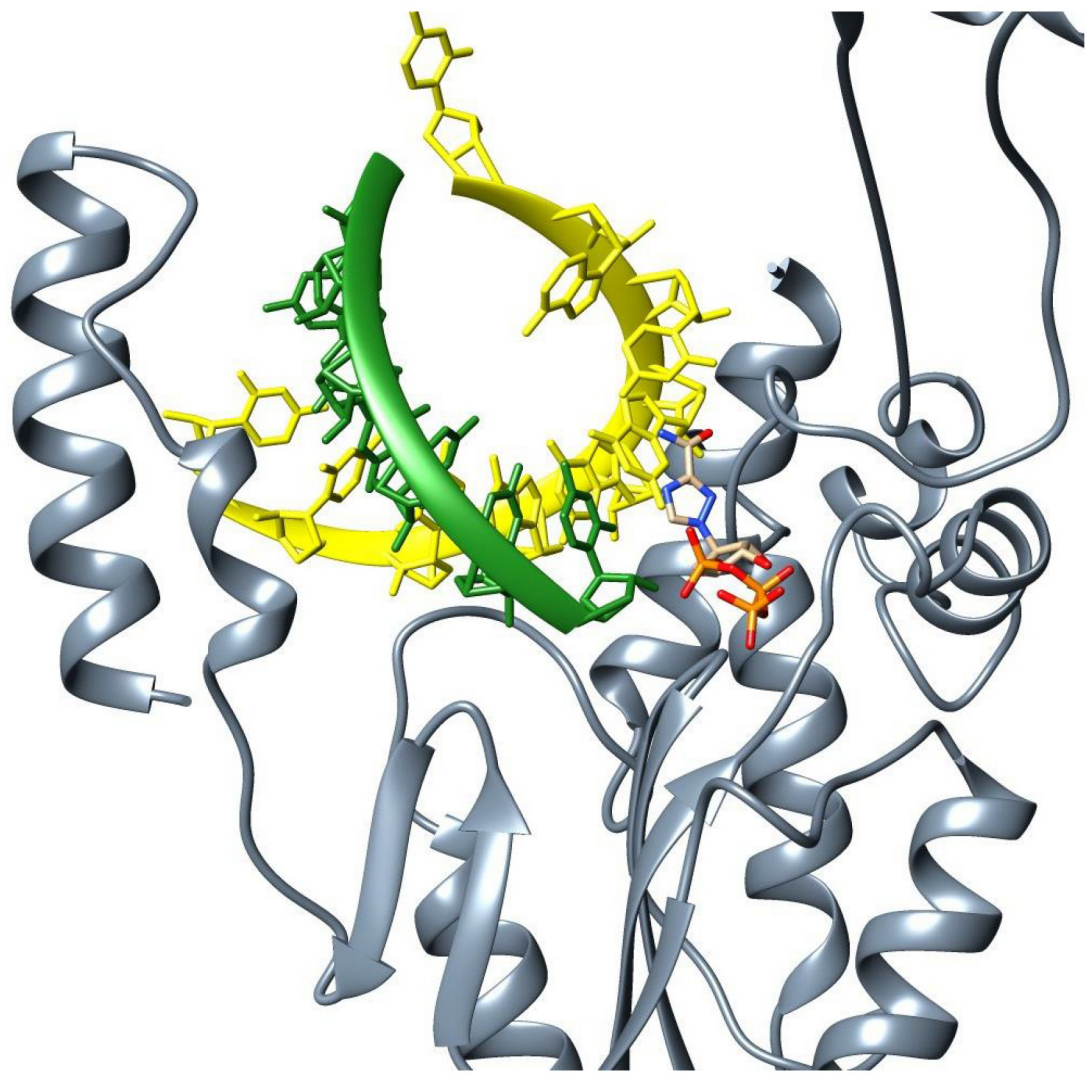
Ebola virus RNA-dependent RNA polymerase predicted model bound to a template-primer RNA and ribavirin triphosphate. The figure is based on the structural superposition with foot-and-mouth disease virus polymerase using the palm subdomain as reference. The active site has been slightly amplified to allow a better visualization. The EBOV polymerase predicted model is in grey, the template strand is in yellow, the primer strand is in green and the ribavirin triphosphate is colored according to Chimera’s elements palette.

A structural superposition of the EBOV RdRp with a foot-and-mouth disease virus polymerase bound to a template-primer RNA and ribavirin triphosphate (Fig 5) was drawn with Chimera 1.8 using the palm subdomain as reference (PDB code 2E9R) [30]. The image shows that the EBOV RdRp central crevice has enough space to hold a dsRNA, and the interactions between the RNA and the protein are analogous to those previously observed in RdRps (Fig 5). The primer might could be bound to residues located in the thumb subdomain and residues within palm domain’s motif E, while the template might be coordinated by residues located in the fingers subdomain. Moreover, the ribavirin triphosphate is located in the predicted active site in which residues from the palm subdomain’s motif B loop and the helix N-terminal region could be interacting with the base-and sugar moieties, and residues from motif A may be forming bonds with the sugar-and triphosphate moieties (S4 Fig). Even though previous work showed that ribavirin had no antiviral effect on animal models [106], our predicted model shows that this drug could fit into the EBOV polymerase NTP binding site, and that most of the interactions of NTPs with the protein could be formed. This supports the work of Morin et al. [36], which demonstrated that ribavirin has *in vitro* activity against the Mononegavirales RdRp.

**Fig 6:**
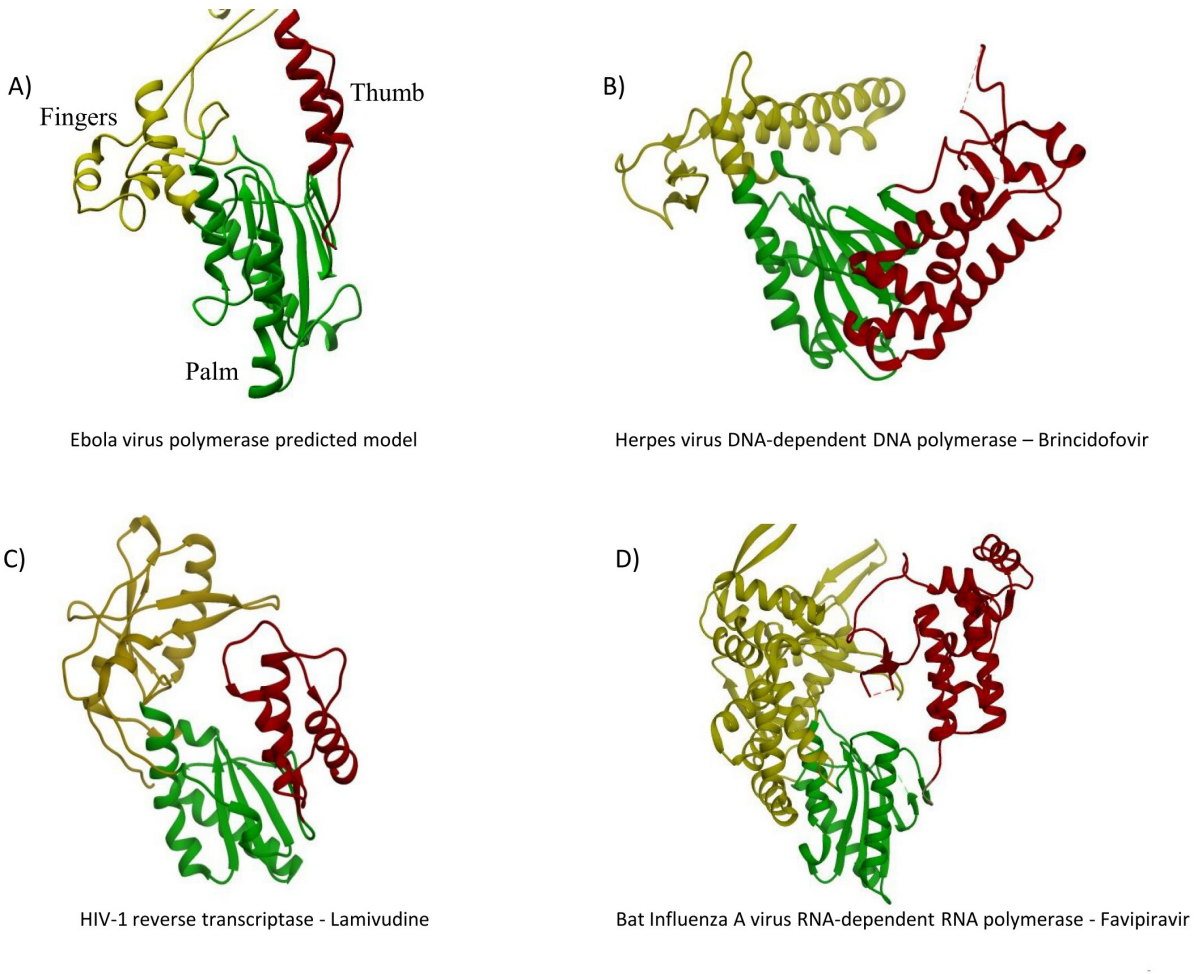
Depiction of the original targets of some of the drugs used against the Ebola virus polymerase. The target polymerase and the name of the drug are indicated below each structure. The three structural subdomains are colored as in Fig 1. (A) Ebola virus polymerase predicted model (B) herpes virus DNA-dependent DNA polymerase (edited from PDB: 2GV9) (C) human immunodeficiency virus-1 reverse transcriptase (edited from PDB: 4G1Q) (D) influenza A virus RNA-dependent RNA polymerase (edited from PDB: 4WSB).

Crystal structures of ss(-)RNA viral polymerases with NTPs at the active site are required in order to elucidate the exact mechanism of nucleotide binding in these enzymes. However, the high level of conservation of the palm subdomain, together with the similarity of the interactions within the active site in DNA- and RNA polymerases are essential to understand why some nucleotide analogues have broad antiviral spectra such as favipiravir, which has proven to have antiviral activity even against different types of RNA viruses, including ss(-) and ss(+)RNA viruses, or brincidofovir, which has antiviral activity against both DNA- and RNA viruses. Our predicted EBOV polymerase model indicates that this RdRp shares the same basic architecture and mechanism of action, including the structural motifs and some of the residues that participate in nucleotide binding. Therefore, drugs aimed at the active site of different types of polymerases (Fig 6), such as those mentioned above, might also interfere with the functionality of the EBOV RdRp, albeit with less specificity.

## Conclusions

RNA-dependent RNA polymerases are encoded by RNA viruses from different families with a variety of genome organization and replication strategies. All known viral RNA polymerases are homologous monomeric enzymes. Whether this is true or not for all RNA viruses remains to be proven. The availability of more than twenty distinct viral RNA polymerase crystals of different RNA viruses and retroviruses reveals the characteristic right hand architecture typical of the superfamily of DNA- and RNA polymerases, with fingers, palm and thumb functional subdomains [4]. The palm subdomain is the catalytic subdomain, and is by far the most conserved region of single subunit DNA- and RNA polymerases [7,9].

Although attempts to group RNA viruses based on polymerases sequence data have been criticized [48], the pioneering analysis of animal and plant viruses by Kamer and Argos [107], Poch et al. [65] and others [108–110] led to the identification of shared conserved motifs and the recognition of the evolutionary relationships between ss(+), ss(-), dsRNA and retroviruses based on RdRp and RT sequences. In this work we have constructed a tertiary structure-based phylogeny that includes viral RdRps and RTs, as well as an eukaryotic telomerase (Fig 2). Our phylogeny exhibits an overall topology similar to those reported by Mönttinen et al. [53] and Cerny et al. [10], although the three trees are based on different assumptions. It has been argued that viral polymerases are not good phylogenetic markers [48]. However, the robustness of three-dimensional based phylogenies is supported by the consistency of the results reported by Cerny et al [10], Mönttinen et al [53] and ourselves, in which the same basic topology and branch distributions are observed. These results indicate that three dimensional-based phylogenies are an important alternative to the primary structure-based phylogenetic trees of RNA-based genetic systems. It is interesting to note that none of the trees is consistent with the Baltimore classification of RNA viruses [54], suggesting the polyphyly of changes in template organization, especially of double-stranded RNA genomes, because of their enhanced chemical stability.

We have also proposed here a three dimensional model of the EBOV RdRp (Fig 3) using homology-based structural prediction of the available amino acid sequences of the Ebola L protein based on the highly conserved and widely distributed motifs characteristic of the polymerase palm domain. These conserved motifs play a critical role in nucleotidyl transfer reaction, ribonucleotide binding, and in the conformational changes of the enzyme. Our predicted fragment of the EBOV polymerase is in agreement with the recently reported structure of the VSV polymerase [40]. The approach we have developed is comparable to the calculation of a model of an Arenavirus RNA polymerase using the hepatitis C viral RNA polymerase reported by Vieth et al [38]. Using as a scaffold the recently reported crystal structure of the bat influenza A viral polymerase, we have developed an *in silico* model of the spatial distribution of a 253-amino acid residue data set of the Ebola virus RNA polymerase with a 92% certainty.

A multiple alignment based on secondary structure prediction of the negative single stranded RNA viruses of the Mononegavirales order (S2 Fig) allowed us to identify several conserved residues, not only in this group of viruses but also in ss(+), ss(-), dsRNA viruses and reverse transcribing viruses. As summarized in this work, our model includes the A-E conserved structural motifs described in other viral RdRps that define the highly conserved right-hand catalytic palm subdomain as well as portions of the fingers and thumb subdomains. The conserved structural similarity of the EBOV polymerase palm subdomain with the viral and cellular DNA polymerases proposed here is consistent with the hypothesis that it is one of oldest identifiable structural domains present in extant viruses and cells [7–9]. The monophyletic origin of all the monomeric polymerases analyzed here has important implications for our understanding of the origin and evolution of mobile genetic elements.

The crystal structures of complexes of DNA- and RNA polymerases with nucleotides or nucleotide analogues show that very similar binding mechanisms are involved [55,101–105]. The incoming nucleotide has several interactions with key residues within the active site in order to be correctly positioned for the nucleophilic attack. The work presented here helps to understand the current use and apparent success of antivirals, i.e. Brincidofovir, Lamivudine and Favipiravir, originally aimed at other types of polymerases, to attack the Ebola virus infection. The strong conservation of the EBOV polymerase functional sites discussed here on the basis of its three-dimensional structure explains the action of these replication inhibitors originally designed for DNA and distinct RNA viruses, and may assist in the search of new therapeutic agents against these subcellular pathogens.

## Supporting Information

S1 Fig Fragment of the bat influenza A virus polymerase used for the EBOV pol model construction. On the left, colored in red, the fragment of the bat influenza A virus polymerase (edited from PDB 4WSB) in which the PHYRE 2.0 web server yielded confidence levels of 91.7% for the EBOV polymerase. On the right, the PHYRE 2.0 sequence alignment for the corresponding fragments of the EBOV and bat influenza A virus polymerases used for the construction of the three-dimensional EBOV polymerase model.
(TIF)

S2 Fig Multiple alignment of RNA-dependent RNA polymerases of viruses that belong to the Mononegavirales order based on a secondary structure prediction. The residues predicted to form helical structures are in red; the residues predicted to form β structures are in blue. Only the residues that could be matched to the bat influenza A virus in the Ebola virus polymerase are shown (vide supra). The lines below the multiple alignment match the polymerases subdomains: fingers subdomain, yellow; palm subdomain, green; thumb subdomain, red. The colored frames correspond to the RNA-dependent RNA polymerases conserved structural motifs: motif A, red; motif B, blue; motif C, green; motif D, magenta; motif E, cyan; motif F, orange. The catalytic aspartic acid residues are highlighted in red. The residues with a high degree of conservation in the Mononegavirales are highlighted in yellow.
(TIF)

S3 Fig Structural superposition of single-stranded negative RNA viral polymerases crystal structures. The figure shows the superposition of the RdRp domains of bat influenza A virus (colored in brown), LaCrosse virus (colored in blue), vesicular stomatitis virus (colored in purple) and our proposal of the EBOV polymerase model (colored in green). The image exhibits the high level of structural conservation in the palm subdomain and the structural motifs of the fingers and thumb subdomains of the polymerases from single-stranded negative RNA viruses. The superposition was created with the MatchMaker program included in Chimera 1.8 [45].
(TIF)

S4 Fig Close-up of the Ebola virus polymerase active site with ribavirin triphosphate. The structural superposition shown here is the same as in Fig 5. The Ebola virus polymerase is colored cyan. The side chains of residues within the structural motifs that might participate in substrate binding have been depicted and colored according to their chemical elements. Ribavirin is colored orange and according to the charge of its chemical elements. The conformation of the rotamers is based on the structural prediction of our Ebola virus polymerase model with the influenza A PB1 protein as a reference.
(TIF)

## References

[1] Hansen JL, Long AM, Schultz SC. Structure of the RNA-dependent RNA polymerase of poliovirus. Structure. 1997: 5:1109–1122. 9309225 10.1016/s0969-2126(97)00261-x

[2] Sesmero E, Thorpe IF. Using the hepatitis C virus RNA-dependent RNA polymerase as a model to understand viral polymerase structure, function and dynamics. Viruses. 2015; 7:3974–3994. 10.3390/v7072808 26193306 PMC4517137

[3] Ng KKS, Arnold JJ, Cameron CE. Structure-function relationships among RNA-dependent RNA polymerases. Curr Top Microbiol Immunol. 2008; 320:137–156. 18268843 10.1007/978-3-540-75157-1_7 PMC2441838

[4] Ortín J, Parra F. Structure and function of RNA replication. Annu Rev Microbiol. 2006; 60:305–326. 16719717 10.1146/annurev.micro.60.080805.142248

[5] Steitz TA. DNA polymerases: structural diversity and common mechanisms. J Biol Chem. 1999; 274:17395–17398. 10364165 10.1074/jbc.274.25.17395

[6] Joyce CM, Steitz TA. Function and structure relationships in DNA polymerases. Annu Rev Biochem. 1994; 63:777–822. 7526780 10.1146/annurev.bi.63.070194.004021

[7] Lazcano A, Fastag J, Gariglio P, Ramírez C, Oró J. On the early evolution of RNA polymerase. J Mol Evol.1988; 27: 365–376 3146647 10.1007/BF02101199

[8] García-Meza V, González Rodríguez A, Lazcano A. Ancient paralogous gene duplications and the search for Archean cells In: Fleischaker GR, Colonna S, Luisi PL, editors. Self-Reproduction of Supramolecular Structures: from synthetic structures to models of minimal living systems. Kluwer Academic Publishers, Dordrecht 1994: 231–246

[9] Delaye L, Vázquez H, Lazcano A. The cenancestor and its contemporary biological relics: the case of nucleic acid polymerases In: Chela-Flores J., Owen T., and Raulin F., editors. First steps in the origin of life in the Universe: Proceedings of the Sixth Trieste Conference on Chemical Evolution. Kluwer Academic Publisher, Dordrecht 2001: 223–230.

[10] Cerny J, Cerna Bolfıkova B, Valdes JJ, Grubhoffer L, Ruzek D. Evolution of tertiary structure of viral RNA dependent polymerases. PLoS ONE. 2014 (5):e96070 10.1371/journal.pone.0096070 24816789 PMC4015915

[11] Ferrer-Orta C, Arias A, Escarmis C, Verdaguer N. A comparison of viral RNA-dependent RNA polymerases. Curr Opin Struct Biol. 2006; 16:27–34. 16364629 10.1016/j.sbi.2005.12.002

[12] Lesburg CA, Cable MB, Ferrari E, Hong Z, Mannarino AF, Weber PC. Crystal structure of the RNA-dependent RNA polymerase from hepatitis C virus reveals a fully encircled active site. Nat Struct Biol. 1999; 6:937–943. 10504728 10.1038/13305

[13] Gorbalenya AE, Pringle FM, Zeddam JL, Luke BT, Cameron CE, Kalmakoff J, et al The palm subdomain-based active site is internally permuted in viral RNA-dependent RNA polymerases of an ancient lineage. J Mol Biol. 2002; 324:47–62. 12421558 10.1016/S0022-2836(02)01033-1 PMC7127740

[14] Pan J, Vakharia VN, Tao YJ. The structure of a birnavirus polymerase reveals a distinct active site topology. Proc Natl Acad Sci U.S.A.2007; 166:1–6. 10.1073/pnas.0611599104 PMC1855279 17456597

[15] Gerlach P, Malet H, Cusack S, Reguera J. Structural insights into Bunyavirus replication and its regulation by the vRNA promoter. Cell. 2015; 161:1–13. 10.1016/j.cell.2015.05.006 PMC4459711 26004069

[16] Gohara DW, Arnold JA, Cameron CE. Poliovirus RNA-dependent RNA polymerase (3Dpol): kinetic, thermodynamic and structural analysis of ribonucleotide selection. Biochemistry. 2004; 43:5149–5158. 15122880 10.1021/bi035429s PMC2426919

[17] Pflug A, Guilligay D, Reich S, Cusack S. Structure of Influenza A polymerase bound to the viral RNA promoter. Nature. 2014; 10.1038/nature14008 25409142

[18] Joyce CM. Choosing the right sugar: how polymerases select nucleotide substrate? Proc Natl Acad Sci U.S.A. 1997; 94:1619–1622. 9050827 10.1073/pnas.94.5.1619 PMC34142

[19] Brown JA, Suo Z. Unlocking the sugar “steric gate” of DNA polymerases. Biochemistry. 2011; 50:1135–1142. 10.1021/bi101915z 21226515 PMC3040255

[20] Garriga D, Ferrer-Orta C, Querol-Audi J, Oliva B, Verdaguer N. Role of motif B loop in allosteric regulation of RNA-dependent RNA polymerization activity. J Mol Biol. 2013; 425:2279–2287. 10.1016/j.jmb.2013.03.034 23542342

[21] Lang DM, Zemla AT, Ecale Zhou CL. Highly similar structural frames link the template tunnel and NTP entry tunnel to the exterior surface in RNA-dependent RNA polymerases. Nucleic Acids Res. 2013; 41:1464–1482. 10.1093/nar/gks1251 23275546 PMC3561941

[22] Sankar S, Porter AG. Point mutations which drastically affect the polymerization activity of encephalomyocarditis virus RNA-dependent RNA polymerase correspond to the active site of Escherichia coli DNA polymerase I. J Biol Chem. 1992;267:10168–10176. 1315753

[23] Reich S, Guilligay D, Pflug A, Malet H, Berger I, Crepin T, et al Structural insight into cap-snatching and RNA synthesis by influenza polymerase. Nature. 2014; 10.1038/nature14009 25409151

[24] Lohmann V, Korner F, Herian U, Bartenschlager R. Biochemical properties of hepatitis C virus NS5B RNA-dependent RNA polymerase and identification of amino acid sequence motifs essential for enzymatic activity. J Virol. 1997; 71:8416–8428. 9343198 10.1128/jvi.71.11.8416-8428.1997 PMC192304

[25] López-Vázquez A, Martín Alonso JM, Parra F. Mutation analysis of the GDD sequence motif of a Calicivirus RNA-Dependent RNA polymerase. J Virol. 2000; 74:3888–3891. 10729164 10.1128/jvi.74.8.3888-3891.2000 PMC111898

[26] Cameron CE, Moustafa IM, Arnold JJ. Dynamics: the missing link between structure and function of the viral RNA-dependent RNA polymerase? Curr Opin Struct Biol. 2009; 19:768–774. 10.1016/j.sbi.2009.10.012 19910183 PMC2787719

[27] Yang X, Smidansky ED, Maksimchuk KR, Lum D, Welch JL, Arnold JJ, et al Motif D of viral RNA-dependent RNA polymerases determines efficiency and fidelity of nucleotide addition. Structure. 2012; 20:1519–1527. 10.1016/j.str.2012.06.012 22819218 PMC3438331

[28] Castro C, Smidansky ED, Arnold JJ, Maksimchuk KR, Moustafa I, Uchida A, et al Nucleic acid polymerases use a general acid for nucleotidyl transfer. Nat Struct Mol Biol. 2009; 16:212–8. 10.1038/nsmb.1540 19151724 PMC2728625

[29] Jacobo-Molina A, Ding J, Nanni RG, Clark AD Jr, Lu X, Tantillo C, et al Crystal structure of human immunodeficiency virus type 1 reverse transcriptase complexed with double-stranded DNA at 3.0 A resolution shows bent DNA. Proc. Natl. Acad. Sci. USA. 1993; 90:6320–6324. 7687065 10.1073/pnas.90.13.6320 PMC46920

[30] Ferrer-Orta C, Arias A, Pérez-Luque R, Escarmis C, Domingo E, Verdaguer N. Sequential structures provide insights into the fidelity of RNA replication. Proc. Natl. Acad. Sci. USA. 2007; 104:9463–9468. 17517631 10.1073/pnas.0700518104 PMC1890517

[31] Appleby TC, Luecke H, Hoon Shim J, Wu JZ, Wayne Cheney I, Zhong W, et al Crystal structure of complete rhinovirus RNA polymerase suggests front loading of protein primer. J Virol. 2005; 79:277–288. 15596823 10.1128/JVI.79.1.277-288.2005 PMC538717

[32] Choi KH, Groarke JM, Young DC, Kuhn RJ, Smith JL, Pevear DC, et al The structure of the RNA-dependent RNA polymerase from bovine viral diarrhea virus establishes the role of GTP in *de novo* initiation. Proc Natl Acad Sci U S A. 2004; 101:4425–4430. 15070734 10.1073/pnas.0400660101 PMC384763

[33] Wu J, Liu W, Gong P. A Structural Overview of RNA-Dependent RNA Polymerases from the Flaviviridae Family. Int J Mol Sci. 2015; 16:12943–12957. 10.3390/ijms160612943 26062131 PMC4490480

[34] Butcher SJ, Grimes JM, Makevey EV, Bamford DH, Stuart DI. A mechanism for initiating RNA-dependent RNA polymerization. Nature. 2001; 410:235–240 11242087 10.1038/35065653

[35] Noton SL, Cowton VM, Zack CR, McGivern DR, Fearns R. Evidence that the polymerase of respiratory syncytial virus initiates RNA replication in a nontemplated fashion. Proc Nat Acad Sci USA. 2010; 107:10226–10231. 10.1073/pnas.0913065107 20479224 PMC2890450

[36] Morin B, Rahmeh AA, Whelan SPJ. Mechanism of RNA synthesis initiation by the vesicular stomatitis virus polymerase. EMBO J. 2012; 31:1320–1329. 10.1038/emboj.2011.483 22246179 PMC3297992

[37] Hass M, Lelke M, Busch C, Becker-Ziaja B, Günther S. Mutational evidence for a structural model of the Lassa virus RNA polymerase domain and identification of two residues, Gly1394 and Asp1395, that are critical for transcription but not replication of the genome. J Virol. 2008; 82:10207–17. 10.1128/JVI.00220-08 18667512 PMC2566270

[38] Vieth S, Torda AE, Asper M, Schmitz H, Gunther S. Sequence analysis of L RNA of Lassa virus. Virology. 2004; 318:153–168. 14972544 10.1016/j.virol.2003.09.009

[39] Kuhn JH. Proposal for a revised taxonomy of the family Filoviridae: classification, names of taxa and viruses, and virus abbreviations. Arch Virol. 2010; 155:2083–2103. 10.1007/s00705-010-0814-x 21046175 PMC3074192

[40] Liang B, Li Z, Jenni S, Rahmeh AA, Morin BM, Grant T, et al Structure of the L protein of vesicular stomatitis virus from electron cryomicroscopy. Cell. 2015; 162:1–14. 10.1016/j.cell.2015.06.018 PMC4557768 26144317

[41] Krissinel E, Henrick K. Secondary-structure matching (PDBeFold), a new tool for fast protein structure alignment in three dimensions. Acta Cryst. 2004; D60:2256–2268. 10.1107/S0907444904026460 15572779

[42] Subbiah S, Laurents DV, Levitt M. Structural similarity of DNA-binding domains of bacteriophage repressors and the globin core. Current Biology. 1993; 3:141–148 15335781 10.1016/0960-9822(93)90255-m

[43] Kelley LA, Mezulis S, Yates CM, Wass MW, Sternberg MJE. The Phyre2 web portal for protein modeling, prediction and analysis. Nature Protocols. 2015; 10:845–858.0 10.1038/nprot.2015.053 25950237 PMC5298202

[44] Marchler-Bauer A, Zheng C, Chitsaz F, Derbyshire MK, Geer LY, Geer RC, et al CDD: conserved domains and protein three-dimensional structure. Nucl Acids Res. 2013; 41(D1):D348–52. 23197659 10.1093/nar/gks1243 PMC3531192

[45] Pettersen EF, Goddard TD, Huang CC, Couch GS, Greenblatt DM, Meng EC, et al UCSF Chimera: a visualization system for exploratory research and analysis. J Comput Chem. 2004; 25:1605–12. 15264254 10.1002/jcc.20084

[46] Pei J, Kim BH, Grishin NV. PROMALS3D: a tool for multiple sequence and structure alignment. Nucleic Acids Res. 2008; 36:2295–2300. 10.1093/nar/gkn072 18287115 PMC2367709

[47] Koonin E. The phylogeny of RNA-dependent RNA polymerases of positive-strand RNA viruses. J Gen Virol. 1991; 72:2197–2206. 1895057 10.1099/0022-1317-72-9-2197

[48] Zanotto PMA, Gibbs MJ, Gould EA, Holmes EC. A reevaluation of the higher taxonomy of viruses based on RNA polymerases. J Virol. 1996; 70:6083–6096. 8709232 10.1128/jvi.70.9.6083-6096.1996 PMC190630

[49] Kormelink R, García ML, Goodin M, Sasaya T, Haenni AL. Negative-strand RNA viruses: the plant-infecting counterparts. Virus Res. 2011; 162:184–202. 10.1016/j.virusres.2011.09.028 21963660

[50] Mayr G, Domingues FS, Lackner P. Comparative analysis of protein structural alignments. BMC Struct Biol. 2007; 7:50–65. 17672887 10.1186/1472-6807-7-50 PMC1959231

[51] Dias A, Bouvier D, Crépin T, McCarthy AA, Hart DJ, Baudin F, et al The cap-snatching endonuclease of influenza virus polymerase resides in the PA subunit. Nature. 2009; 458:914–918. 10.1038/nature07745 19194459

[52] Reguera J, Weber F, Cusack S. Bunyaviridae RNA polymerases (L-Protein) have an N-Terminal, Influenza-like endonuclease domain, essential for viral cap-dependent transcription. PLoS Pathog. 2010; 6(9):e1001101 10.1371/journal.ppat.1001101 20862319 PMC2940753

[53] Mönttinen HA, Ravantti JJ, Stuart DI, Poranen MM. Automated structural comparisons clarify the phylogeny of the right-hand-shaped polymerases. Mol Biol Evol. 2014; 31: 2741–2752 10.1093/molbev/msu219 25063440

[54] Baltimore D. Expression of animal viral genomes. Bacteriol Rev. 1971; 35: 235–241. 4329869 10.1128/br.35.3.235-241.1971 PMC378387

[55] Johnson KM. Ebola haemorrhagic fever in Zaire, 1976. Bull World Health Organ. 1978; 56:271–293. 307456 PMC2395567

[56] Baron RC, McCormick JB, Zubeir OA. Ebola virus disease in Sudan: hospital dissemination and intrafamilial spread. Bull World Health Organ.1983; 61:997–1003. 6370486 PMC2536233

[57] MacNeil A, Farno EC, Wamala J, Okware S, Cannon DL, Reed Z, et al Proportion of deaths and clinical features in Bundibugyo Ebola virus infection, Uganda. Emerg Infect Dis. 2010; 16:1969–1972. 10.3201/eid1612.100627 21122234 PMC3294552

[58] Pringle CR, Easton AJ. Monopartite negative strand RNA genomes. Semin Virol. 1997; 8:49–57.

[59] Muhlberger E. Filovirus replication and transcription. Future Virol. 2007; 2:205–215. 24093048 10.2217/17460794.2.2.205 PMC3787895

[60] Huang Y, Xu L, Sun Y, Nabel GL. The assembly of Ebola Virus nucleocapsid requires virion-associated proteins 35 and 24 and posttranslational modification of nucleoprotein. Mol Cell. 2002; 10:307–316 12191476 10.1016/s1097-2765(02)00588-9

[61] Han Z, Boshra H, Sunyer JO, Zwiers SH, Paragas J, Harty RN. Biochemical and functional characterization of the Ebola virus VP24 protein: implications for a role in virus assembly and budding. J Virol. 2003; 77:1793–1800. 12525613 10.1128/JVI.77.3.1793-1800.2003 PMC140957

[62] Reid SP, Leung LW, Hartman AL, Martínez O, Shaw ML, Carbonelle C, et al Ebola virus VP24 binds Karyopherin α1 and blocks STAT1 nuclear accumulation. J Virol. 2006; 80:5156–5167. 16698996 10.1128/JVI.02349-05 PMC1472181

[63] Volchkov VE, Volchkova VA, Chepurnov AA, Blinov VM, Dlonik O, Netesov SV, et al Characterization of the L gene and 5’trailer region of Ebola virus. J Gen Virol. 1999; 80:355–362. 10073695 10.1099/0022-1317-80-2-355

[64] Barr JN, Wertz GG. Polymerase slippage at vesicular stomatitis virus gene junctions to generate Poly(A) is regulated by the upstream 3′-AUAC-5′ tetranucleotide: implications for the mechanism of transcription termination. J Virol. 2001; 75:6901–6913. 11435570 10.1128/JVI.75.15.6901-6913.2001 PMC114418

[65] Poch O, Blumberg BM, Bougeueleret L, Tordo N. Sequence comparison of five polymerases (L proteins) of unsegmented negative-strand RNA viruses: theoretical assignment of functional domains. J Gen Virol. 1990; 71:1153–1162. 2161049 10.1099/0022-1317-71-5-1153

[66] Bruenn JA. A structural and primary sequence comparison of the viral RNA-dependent RNA polymerases. Nucleic Acids Res. 2003; 31:1821–1829. 12654997 10.1093/nar/gkg277 PMC152793

[67] Schnell MJ, Conzelmann KK. Polymerase activity of in vitro mutated Rabies virus L protein. Virology. 1995; 214:522–530. 8553554 10.1006/viro.1995.0063

[68] Malur AG, Gupta NK, De Bishnu P, Banerjee AK. Analysis of the mutations in the active site of the RNA-dependent RNA polymerase of human parainfluenza virus type 3 (HPIV3). Gene Expr. 2002; 10:93–100. 12064576 PMC5977508

[69] Chattopadhyay A, Raha T, Shaila MS. Effect of single amino acid mutations in the conserved GDNQ motif of L protein of Rinderpest virus on RNA synthesis in vitro and in vivo. Virus Res. 2004; 99:139–145. 14749179 10.1016/j.virusres.2003.11.003

[70] Rosenberg R. Detecting the emergence of novel, zoonotic viruses pathogenic to humans. Cell Mol Life Sci. 2014; 10.1007/s00018-014-1785-y PMC4629502 25416679

[71] De Clerq E. A cutting-edge view on the current state of antiviral drug development. Med Res Rev. 2013; 6:1249–1277. 10.1002/med.21281 23495004

[72] Bray M. Highly pathogenic RNA viral infections: challenges for antiviral research. Antivir Res. 2008; 78: 1–8. 10.1016/j.antiviral.2007.12.007 18243346

[73] Bishop BM. Potential and emerging treatment options for Ebola virus disease. Ann Pharmacother. 2014; 10.1177/1060028014561227 25414384

[74] Oestereich L, Ludtke A, Wurr S, Rieger T, Muñoz-Fontela C, Gunther S. Successful treatment of advanced Ebola virus infection with T-705 (favipiravir) in a small animal model. Antiviral Res. 2014; 105:17–21. 10.1016/j.antiviral.2014.02.014 24583123

[75] Fauci AS, Collins FS. NIH Ebola Update: Working Toward Treatments and Vaccines. October 14^th^, 2014. Available: http://directorsblog.nih.gov/2014/10/14/nih-ebola-update-working-toward-treatments-and-vaccines/

[76] Magee WC, Hostetler KY, Evans DH. Mechanism of inhibition of vaccinia virus DNA polymerase by cidofovir diphosphate. Antimicrob Agents Chemother. 2005; 49:3153–3162. 16048917 10.1128/AAC.49.8.3153-3162.2005 PMC1196213

[77] Olson VA, Smith SK, Foster S, Li Y, Lanier R, Gates I, et al *In Vitro* efficacy of Brincidofovir against variola virus. Antimicrob Agents Chemother. 2014; 58:5570–5571. 10.1128/AAC.02814-14 24957837 PMC4135866

[78] Sandkovsky U, Vargas L, Florescu DF. Adenovirus: current epidemiology and emerging approaches to prevention and treatment. Curr Infect Dis Rep. 2014; 16:416–423. 10.1007/s11908-014-0416-y 24908344

[79] Painter W, Robertson A, Trost LC, Godkin S, Lampert B, Painter G. First pharmacokinetic and safety study in humans of the novel lipid antiviral conjugate CMX001, a broad-spectrum oral drug active against double-stranded DNA viruses. Antimicrob Agents Chemother. 2012; 56:2726–34. 10.1128/AAC.05983-11 22391537 PMC3346600

[80] Marty FM, Winston DJ, Rowley SD, Vance E, Papanicolaou GA, Mullane KM, et al CMX001 to prevent Cytomegalovirus disease in hematopoietic-cell transplantation. N Engl J Med. 2013; 369:1227–1236. 10.1056/NEJMoa1303688 24066743

[81] Clair MH St, Richards CA, Spector T, Weinhold KJ, Miller WH, Langlois AJ, et al 3'-Azido-3'-deoxythymidine triphosphate as an inhibitor and substrate of purified human immunodeficiency virus reverse transcriptase. Antimicrob Agents Chemother. 1987; 31:1972–1977. 2449866 10.1128/aac.31.12.1972 PMC175837

[82] Sherman M, Shafran S, Burak K, Doucette K, Wong W, Girgrah N, et al Management of chronic hepatitis B: Consensus guidelines. Can J Gastroenterol. 2007; 21 (Suppl C): 5C–24C. 17568823 PMC2794455

[83] Larder BA, Kemp SD, Harrigan PR. Potential mechanism for sustained antiretroviral efficacy of AZT-3TC combination therapy. Science. 1995; 269:696–699. 7542804 10.1126/science.7542804

[84] Azango M. Liberian Doctor Defends 3–5 Days Ebola Treatment With HIV Drug. Published on September 29^th^, 2014. Available: http://allafrica.com/stories/201409292050.html

[85] Furuta Y, Gowen BB, Takahashi K, Shiraki K, Smee DF, Barnard DL. Favipiravir (T-705), a novel RNA polymerase inhibitor. Antiviral Res. 2013; Available: 10.1016/j.antiviral.2013.09.015 PMC3880838 24084488

[86] Furuta Y, Takahashi K, Fukuda Y, Kuno M, Kamiyama T, Kozaki K, et al *In vitro* and *in vivo* activities of anti-influenza virus compound T-705. Antimicrob Agents Chemother. 2002; 46, 977–981 11897578 10.1128/AAC.46.4.977-981.2002 PMC127093

[87] Gowen BB, Wong MH, Jung KH, Sanders AB, Mendenhall M, Bailey KW, et al *In vitro and in vivo* activities of T-705 against arenavirus and bunyavirus infections. Antimicrob Agents Chemother. 2007; 51:3168–3176. 17606691 10.1128/AAC.00356-07 PMC2043187

[88] Gowen BB, Wong MH, Jung KH, Smee DF, Morrey JD, Furuta Y. Efficacy of favipiravir (T-105) and T-1106 pyrazine derivatives in phlebovirus disease models. Antiviral Res. 2010; 86:121–127. 10.1016/j.antiviral.2009.10.015 19874853 PMC3008316

[89] Morrey JD, Taro BS, Siddharthan V, Wang H, Smee DF, Christensen AJ, et al Efficacy of orally administered T-705 pyrazine analog on lethal West Nile virus infection in rodents. Antiviral Res. 2008; 80:377–379. 10.1016/j.antiviral.2008.07.009 18762216 PMC2587511

[90] Julander JG, Shafer K, Smee DF, Morrey JD, Furuta Y. Activity of T-705in a hamster model of yellow fever virus infection in comparison with that of a chemically related compound, T-1106. Antimicrob Agents Chemother. 2009; 53: 202–209. 10.1128/AAC.01074-08 18955536 PMC2612161

[91] Julander JG, Smee DF, Morrey JD, Furuta Y. Effect of T-705 treatment on western equine encephalitis in a mouse model. Antiviral Res. 2009; 82:169–171. 10.1016/j.antiviral.2009.02.201 19428608 PMC2704019

[92] Rocha-Pereira J, Jochmans D, Dallmeier K, Leyssen P, Nascimento MS, Neyts J. Favipiravir (T-705) inhibits in vitro norovirus replication. Biochem Biophys Res Commun. 2012; 424:777–780. 10.1016/j.bbrc.2012.07.034 22809499

[93] Crotty S, Cameron CE, Andino R. RNA virus error catastrophe: direct molecular test by using Ribavirin. Proc Natl Acad Sci USA. 2001; 98:6895–6900. 11371613 10.1073/pnas.111085598 PMC34449

[94] Vivet-Boudou V, Isel C, El Safadi Y, Smyth RP, Laumond G, Moog C, et al Evaluation of anti-HIV-1 mutagenic nucleoside analogues. J Biol Chem. 2015; 290:371–383. 10.1074/jbc.M114.616383 25398876 PMC4281740

[95] Mayhoub AS. Hepatitis C RNA-dependent RNA polymerase inhibitors: A review of structure-activity and resistance relationships; different scaffolds and mutations. Bioorg Med Chem. 2012; 20: 3150–3161. 10.1016/j.bmc.2012.03.049 22516671

[96] Ren J, Stammers DK. Structural basis for drug resistance mechanisms for non-nucleoside inhibitors of HIV reverse transcriptase. Virus Res. 2008; 134:157–170. 10.1016/j.virusres.2007.12.018 18313784

[97] Sarafianos SG, Marchand B, Das K, Himmel D, Parniak MA, Hughes SH, et al Structure and function of HIV-1 reverse transcriptase: molecular mechanisms of polymerization and inhibition. J Mol Biol. 2009; 385:693–713. 10.1016/j.jmb.2008.10.071 19022262 PMC2881421

[98] Lazcano A, Valverde V, Hernández G, Gariglio P, Fox GE, Oró J. On the early emergence of reverse transcription: theoretical basis and experimental evidence. J Mol Evol. 1992,35:524–536. 1282161 10.1007/BF00160213

[99] Llaca V, Silva E, Lazcano A, Rangel LM, Gariglio P, Oró J. In search of the ancestral RNA polymerase: an experimental approach In: Ponnamperuma C. and Eirich F., editors. Prebiological Self Organization of Matter. Deepak Publ., Hampton, VA 1990: 247–260

[100] Ricchetti M, Buc H. E. coli DNA polymerase I as a reverse transcriptase. EMBO J. 1993, 12:387–396. 7679988 10.1002/j.1460-2075.1993.tb05670.x PMC413221

[101] Doublié S, Tabor S, Long AM, Richardson CC, Ellenberger T. Crystal structure of a bacteriophage T7 DNA replication complex at 2.2 A resolution. Nature. 1998; 391:251–258. 9440688 10.1038/34593

[102] Huang H, Chopra R, Verdine GL, Harrison SC. Structure of a covalently trapped catalytic complex of HIV-1 reverse transcriptase: implications for drug resistance. Science. 1998; 282:1669–1675. 9831551 10.1126/science.282.5394.1669

[103] Swan MK, Johnson RE, Prakash L, Prakash S, Aggarwal AK. Structural basis of high fidelity DNA synthesis by yeast DNA polymerase Delta. Nat Struct Mol Biol. 2009; 16:979–986. 10.1038/nsmb.1663 19718023 PMC3055789

[104] Wu Y, Lou Z, Miao Y, Yu Y, Dong H, Peng W, et al Structures of EV71 RNA-dependent RNA polymerase in complex with substrate and analogue provide a drug target against the hand-foot-and-mouth disease pandemic in China. Protein Cell. 2010; 1:491–500 10.1007/s13238-010-0061-7 21203964 PMC4875138

[105] Alam I, Lee JH, Cho KJ, Han KR, Yang JM, Chung MS, et al Crystal structures of murine norovirus-1 RNA-dependent RNA polymerase in complex with 2-thiouridine or ribavirin. Virology. 2012; 426:143–151. 10.1016/j.virol.2012.01.016 22341781

[106] Huggins JW. Prospects for treatment of viral hemorrhagic fevers with ribavirin, a broad-spectrum antiviral drug. Clin Infect Dis. 1989; 11 (Supplement 4): S750–S761. 10.1093/clinids/11.supplement_4.s750 2546248

[107] Kamer G, Argos P. Primary structural comparison of RNA-dependent polymerases form plant, animal and bacterial viruses. Nucleic Acids Res. 1984; 12:7269–7282. 6207485 10.1093/nar/12.18.7269 PMC320156

[108] Haseloff J, Goelet P, Zimmern D, Ahlquist P, Dasgupta R, Kaesberg P. Striking similarities in amino acid sequence among nonstructural proteins encoded by RNA viruses that have dissimilar genomic organization. Proc Natl Acad Sci U.S.A. 1984; 81:4358–4362. 6611550 10.1073/pnas.81.14.4358 PMC345588

[109] Doolittle R, Feng DF, Johnson MS, McClure MA. Origins and evolutionary relationships of retroviruses. Q Rev Biol. 1989, 64:1–30. 2469098 10.1086/416128

[110] Koonin EV, Dolja VV. Evolution and taxonomy of positive-strand RNA viruses: implications of comparative analysis of amino acid sequences. Crit Rev Biochem Mol Biol. 1993, 28:375–430. 8269709 10.3109/10409239309078440

